# Distribution, Amplitude, Incidence, Co-Occurrence, and Propagation of Human K-Complexes in Focal Transcortical Recordings^1^,^2^,^3^

**DOI:** 10.1523/ENEURO.0028-15.2015

**Published:** 2015-09-17

**Authors:** Rachel A. Mak-McCully, Burke Q. Rosen, Matthieu Rolland, Jean Régis, Fabrice Bartolomei, Marc Rey, Patrick Chauvel, Sydney S. Cash, Eric Halgren

**Affiliations:** 1 Department of Neurosciences, University of California, San Diego, San Diego, California 92093; 2 Department of Radiology, University of California, San Diego, San Diego, California 92093; 3 Department of Psychiatry, University of California, San Diego, San Diego, California 92093; 4 Aix-Marseille Université, Marseille 13385, France; 5 INSERM, Institut de Neurosciences des Systèmes UMR 1106, Marseille 13005, France; 6 Assistance Publique–Hôpitaux de Marseille, Timone Hospital, Marseille 13005, France; 7 Department of Neurology, Massachusetts General Hospital and Harvard Medical School, Harvard University, Boston, Massachusetts 02114

**Keywords:** depth recordings, k-complex, memory, SEEG, sleep

## Abstract

K-complexes (KCs) are thought to play a key role in sleep homeostasis and memory consolidation; however, their generation and propagation remain unclear. The commonly held view from scalp EEG findings is that KCs are primarily generated in medial frontal cortex and propagate parietally, whereas an electrocorticography (ECOG) study suggested dorsolateral prefrontal generators and an absence of KCs in many areas. In order to resolve these differing views, we used unambiguously focal bipolar depth electrode recordings in patients with intractable epilepsy to investigate spatiotemporal relationships of human KCs. KCs were marked manually on each channel, and local generation was confirmed with decreased gamma power. In most cases (76%), KCs occurred in a single location, and rarely (1%) in all locations. However, if automatically detected KC-like phenomena were included, only 15% occurred in a single location, and 27% occurred in all recorded locations. Locally generated KCs were found in all sampled areas, including cingulate, ventral temporal, and occipital cortices. Surprisingly, KCs were smallest and occurred least frequently in anterior prefrontal channels. When KCs occur on two channels, their peak order is consistent in only 13% of cases, usually from prefrontal to lateral temporal. Overall, the anterior–posterior separation of electrode pairs explained only 2% of the variance in their latencies. KCs in stages 2 and 3 had similar characteristics. These results open a novel view where KCs overall are universal cortical phenomena, but each KC may variably involve small or large cortical regions and spread in variable directions, allowing flexible and heterogeneous contributions to sleep homeostasis and memory consolidation.

## Significance Statement

This is the first examination of the location of K-complexes (KCs) and their temporal relationship across the cortex using such focal measures of brain activity. KCs, along with sleep spindles and slow oscillations, are thought to play important roles in the restorative and memory consolidation processes of non-rapid eye movement sleep. KCs are unique from these other markers because they indicate isolated periods of cortical silence. We describe here that KCs occur in parts of the brain previously thought not to generate KCs. We show they may co-occur over small or large parts of the cortex, but do not propagate in a systematic way. This variability could reflect and support consolidation processes devoted to memories involving the corresponding cortical areas and sequences.

## Introduction

K-complexes (KCs) are isolated down states occurring both spontaneously and in response to sensory stimuli during non-rapid eye movement (NREM) sleep (Colrain, 2005; Cash et al., 2009). Here we examine the spatial distribution and temporal dynamics of KCs across the cortex using bipolar stereoencephalographic (SEEG) recordings to address basic questions that are unanswered or ambiguous in the KC literature. Which cortical regions generate KCs? How variable is KC amplitude? How variable is the KC occurrence rate? How often do KCs co-occur across the cortex? Do KCs propagate sequentially from one cortical location to another?

Answers to some of these questions have varied due to the recording technique. Scalp EEG examination of the KC indicates that KCs are largest in amplitude and most frequent over midline frontal sites (Colrain, 2005; Halász, 2005). Cortical surface electrocorticography (ECOG) recordings and average reference SEEG recordings have led to the conclusion that many cortical areas crucial for memory do not generate KCs (Wennberg, 2010). Scalp EEG and ECOG recordings, however, are difficult to interpret due to potential overlap and cancellation from multiple distributed generators. Additionally, average references may detect signals that are not locally generated.

Bipolar SEEG recordings eliminate the ambiguity of scalp EEG, ECOG, and average reference SEEG. In both scalp EEG and ECOG, individual electrodes record activity from local and distant generators, which are difficult to disentangle; scalp EEG is further smeared by the skull. In bipolar SEEG recordings, nearby contacts along an electrode are subtracted from one another. Adjacent contacts chosen for analysis can have one contact just above the gray matter in the CSF, and one just below the gray matter in the white matter. This subtraction provides a focal recording of brain activity unavailable with scalp EEG or ECOG. Even the use of an average reference in SEEG may mask or augment a local signal, which is not the case when SEEG is analyzed using bipolar subtraction. The ambiguity inherent in scalp EEG, ECOG, and referential SEEG may confound interpretations of both where KCs are generated and how they co-occur or spread across the cortex.

Another source of ambiguity in previous studies is the grouping together of KCs and slow oscillations (SOs), which occur as a series of up and down states in NREM stage 3 sleep (N3). SOs and KCs share a frequency of ∼1 Hz, and the KC is identical to the down state of the SO (Cash et al., 2009). The commonly used automated SO detection methods identify all large low-frequency deflections, including KCs. Scalp EEG recordings have led to the interpretation that SOs propagate across the cortex in an anterior-to-posterior direction, with a delay of ∼200 ms (Massimini et al., 2004; Murphy et al., 2009). Since these studies included KCs as well as SOs in their analyses, these studies provide insight into the propagation of KCs as well. A major distinction between SOs and KCs is that the KC is an isolated event: it is neither part of an ongoing oscillation nor is preceded by an up state. It is, therefore, possible to pinpoint the start of a KC in the cortex. In contrast, because the SO is an oscillation, it can be difficult to determine which waves correspond to each other across different cortical areas, resulting in ambiguous order and dependence.

By rigorously quantifying the spatial distribution and temporal dynamics of KCs across the cortex, we focused on three questions. First, are KCs a fundamental neocortical state that are locally generated in all neocortical areas with similar amplitudes and rates of occurrence? Particular attention was paid to the cingulate, ventral temporal, and occipital cortices where Wennberg (2010) failed to find KC generators using intracranial recordings. Similarly, special attention was paid to lateral cortex, in general, where other studies using high-density EEG failed to find generators of evoked KCs or isolated SOs (i.e., KCs; Murphy et al., 2009; Riedner et al., 2011). Second, do KCs co-occur in multiple lobes and both hemispheres, or do they occur in a more isolated fashion? Third, do KCs systematically propagate from anterior to posterior locations, and in particular from prefrontal to parietal?

The three NREM sleep markers—sleep spindles, SOs, and KCs—have all been implicated in the restorative and memory processes that occur during sleep (Diekelmann and Born, 2010; Tononi and Cirelli, 2014). By understanding where the KC occurs in the brain, how the KC occurs temporally across the cortex, and whether it is an event that may manifest heterogeneously each time it occurs, we can understand how the role of each generating structure and their orchestration lead to sleep homeostasis and memory consolidation.

## Materials and Methods

SEEG recordings were obtained in nine patients (eight women, one man; mean age, 41.4 ± 4.9 years) who experienced pharmacoresistant epilepsy to localize their seizure focus prior to possible resection at Massachusetts General Hospital or La Timone Hospital (Table 1). The anatomical nomenclature follows that of Destrieux et al. (2010). At Massachusetts General Hospital, electrodes were localized with respect to anatomical structures using computed tomography (CT) with the electrodes in place, and intraoperative photographs (Dykstra et al., 2012). Depth electrodes (SEEG) had either eight contacts with 5 mm center-to-center spacing or six contacts with 8 mm center-to-center spacing. Each contact was 2.4 mm long with a diameter of 1.28 mm. The electrodes usually passed approximately perpendicular to the midsagittal plane. At La Timone Hospital, electrodes were localized using the fusion of CT scanning with implanted electrodes and MRI. In one case, surgical planning and preoperative MRI were used to localize the electrodes. SEEG electrodes had 10 or 15 contacts with 3.5 mm center-to-center spacing; in some cases, the 15 contact electrodes contained three sets of five contacts, with 3.5 mm center-to-center spacing within the set and 7 or 11 mm between each set of five contacts. Contacts were 2 mm long with a 0.8 mm diameter. Electrodes were both perpendicular and oblique. Prior to surgery, fully informed consent was obtained under the auspices of local institutional review boards. The signals were sampled at 256, 500, 512, or 1024 Hz.

**Table 1.** Demographic and clinical information for all patients studied

Subject	Gender	Age (years)	Handedness	Clinical diagnosis	Pathologic diagnosis	Imaging	Focus	IQ (FSIQ)
1	M	45	L	CPS; bitemporal	No pathology obtained		Left and right mesial temporal lobes	83
2	F	58	R	CPS; possible generalized epilepsy	No pathologyobtained		Likely generalized	80
3	F	45	R	CPS; multifocal	Multifocal: temporal, parietal, occipital		Temporal	Average
4	F	65	R	CPS; temporal lobe epilepsy with two foci: left mesial temporal structures and right subfrontal region	No pathology obtained		Right subfrontal and anterior temporal	101
5	F	27	R	Right temporal lobe epilepsy	No pathology obtained	Right hippocampal sclerosis	Hippocampus, entorhinal cortex, amygdala	Average
6	F	32	R	Left temporal lobe epilepsy	Non-specific gliosis	Normal	Hippocampus, entorhinal cortex, amygdala	77
7	F	23	R	Right temporal lobe epilepsy	Type I focal dysplasia	Normal	Hippocampus, entorhinal cortex,anterior insula	86
8	F	28	R	Right temporal occipital epilepsy	Type II focal dysplasia	Focal cortical dysplasia in right fusiform	Fusiform gyrus, entorhinal cortex	Average
9	F	50	R	Right temporal occipital epilepsy	No pathology obtained	Normal	Right fusiform gyrus	110

FSIQ, Full Scale Intelligence Quotient; CPS, Complex Partial Seizures.

Adjacent contacts along each SEEG electrode were subtracted to create bipolar contacts. The bipolar derivation was obtained by subtracting the more lateral lead from the more medial adjacent lead. In the case where the electrode is passing through the most superficial cortex, the lateral lead is over the pia and the medial lead is in the underlying white matter. Since the KC is surface negative (Cash et al., 2009), a medial − lateral derivation would in this case result in a positive peak. In contrast, at the midline locations (e.g., the crown of the cingulate gyrus), the medial lead is above the pia and the lateral is in the underlying white matter, and consequently KCs are negative in the medial − lateral derivation. Intermediate bipolar pairs could be either polarity depending upon the exact relationship of the contacts to local cortical folding.

Contacts to include for analysis were chosen by examining both physiological and anatomical criteria. Physiologically, referential recordings from a given depth electrode were examined for successive contacts which recorded polarity-inverted spontaneous activity. In such cases, anatomically, one contact of the bipolar pair would typically lay in the CSF above the cortical gray matter, while the other lay just below it in the white matter. In the case of overlapping bipolar contacts (i.e., two pairs sharing a contact), only one bipolar pair was included for analysis. A total of 55 bipolar contacts were analyzed. This bipolar method is free of volume conduction and reference lead issues, obtaining unambiguously focal cortical recordings. Furthermore, to be included for analysis, contacts needed to be distant from the epileptogenic focus, free of epileptiform activity (spikes or pathological slowing), and exhibit KCs.

Usable scalp EEG channels were recorded in the four subjects from Massachusetts General Hospital, but not in the five subjects from La Timone Hospital. When present, the scalp recordings lacked the full montage used for formal polysomnography (Silber et al., 2007). Periods when patients were behaviorally noted to be sleeping were selected for analysis. As this study sought to characterize KCs as measured locally, rather than defined by scalp EEG, the initial selection of KCs was blind to sleep staging. As a secondary analysis, sleep staging based on a scalp EEG—as well as EMG and EOG, when available—for subjects recorded at Massachusetts General Hospital, or based on the SEEG for subjects recorded at La Timone Hospital, was performed for all subjects by a qualified rater (M. Rey).

KCs were manually marked independently on each bipolar SEEG contact for each subject. Each night was considered independent and studied separately. Selected KCs were isolated (i.e., they were not part of a preceding oscillation) and exhibited a multiphasic morphology, with a significant drop peaking at ∼500 ms from the beginning of the waveform, often followed by a rebound peaking at ∼900 ms (Colrain, 2005). Each channel was then bandpassed from 0.1 to 5 Hz in order to select the peak of the manually marked KC.

To confirm that selected KCs, regardless of their polarity, were down states, we tested for a drop in high gamma power (HGP) at the time of the KC in all channels for each subject. If a subject had multiple nights, only one sample night was used to test an HGP drop in all channels. KCs were therefore not validated individually for a drop in HGP, but were validated over all KCs in each channel, for 1 night per subject. HGP was calculated from 60 to 120 Hz, or from 70 to 120 Hz, depending on whether the line noise was at 50 or 60 Hz, respectively. Visually, a drop in HGP up to 120 Hz was determined by a time frequency analysis using default wavelets in EEGLAB (Delorme and Makeig, 2004): with the most negative peak of the KC at time zero, −1.5 to −0.5 s was used as a comparison baseline for a *t* test at a *p* < 0.01 uncorrected threshold. The drop in HGP seen at the time of the KC was further verified using a Hilbert transform applied to each KC. The analytic amplitude of the Hilbert transform was calculated after applying a bandpass filter for the appropriate HGP range (either 60–120 Hz or 70–120 Hz) and averaged over all KCs for each channel. Compared to a baseline period from −1.5 to −1 s prior to the most negative peak of the KC at time zero, periods of significant (*p* < 0.01, *t* test, false discovery rate corrected) drop in HGP were required for KC confirmation. These analyses were accomplished with custom MATLAB (2009) routines with dependencies including the FieldTrip (Oostenveld et al., 2011) toolbox.

In order to compare our bipolar derivations with the common average reference recordings used in a previous study (Wennberg, 2010), referential recordings were reconstructed for the individual leads used in a typical transcortical bipolar pair, and in nearby leads on the same probe but in the white matter. HGP was calculated as above (see Fig. 3 for details).

KC detections were grouped across channels to classify the number of unique KC events for each night for each subject using R (R Development Core Team, 2004). Starting with the first KC peak, additional channels were classified as participating in the same KC if their peak time was ≤200 ms from this first peak latency. The next KC peak after the 200 ms window was the beginning of the second KC event. The grouping of KC events continued until the last KC peak was classified. As a control, KCs were also regrouped using a 200 ms crawling window: after each KC peak detection, the detection window was shifted 200 ms to the right. The detection window continued shifting to the right until no more peaks were detected. These regroupings were not used for any of the analyses described below.

Although the 200 ms window procedure would allow KCs occurring in at least two channels to have a maximum peak difference up to 200 ms, the actual maximum difference was observed to be much less. This latency difference was quantified by calculating the delay between the first channel peak and the last channel peak for each KC event. The mean and SD over all KCs for each night were then calculated.

**Figure 1.**
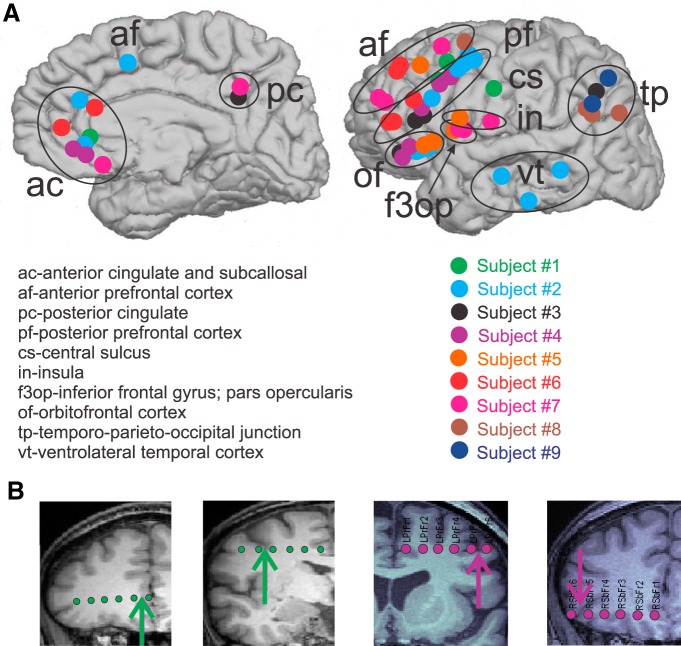
Bipolar SEEG contacts. ***A***, Bipolar SEEG contacts, color coded by subject, are marked by circles on the medial (left) and lateral (right) surfaces of the brain. Bipolar SEEG contacts are grouped into 1 of 10 anatomical groups, as demarcated by black circles and/or one of the following labels: ac (anterior cingulate and subcallosal); af (anterior prefrontal cortex); pc (posterior cingulate); pf (posterior prefrontal cortex); cs (central sulcus); in (insula); f3op (inferior frontal gyrus; pars opercularis); of (orbitofrontal cortex); tp (temporo-parieto-occipital junction); vt (ventrolateral temporal cortex). ***B***, Example SEEG electrodes for subject 1 (green) and subject 4 (purple) are superimposed on the subjects’ MRIs. The arrows indicate the location of the bipolar contact pair chosen on each electrode. From the MRI, it is possible to see that the two contacts in each pair are spanning the local cortical mantle.

To study how KC amplitude and KC occurrence rate may vary across the cortex, bipolar channels were grouped by anatomical locations into 10 groups (Fig. 1). To test KC amplitude, a linear mixed-effects model (Pinheiro et al.,2015) was performed. Location was used as a fixed effect. Placement of electrodes for each patient was chosen to localize the patient’s epilepsy, but electrodes included in this analysis were not shown to be part of each patient’s seizure focus. Therefore, patient and location were considered independent. Interpatient variability was adjusted for by using patient as a random effect. Channel variability due to electrode placement with respect to the surrounding cortex, as well as variable contact spacing, was taken into account by using channel as a random nested effect.

To test the KC occurrence rate, a linear mixed-effects model of the inter-KC interval was performed with location as a fixed effect and patient as a random effect. In both the amplitude and occurrence rate models, the orbitofrontal cortex group served as the reference. For the residuals of the model to be normally distributed, a log transform was applied to the amplitudes and a Box-Cox Transform (Box and Cox, 1964) was applied to the inter-KC intervals. The normality of the residuals was checked visually. All models were developed using the LME procedure implemented in the nlme package (Pinheiro et al., 2015) of R. Due to the limitations of this package, confidence intervals for all predicted values derived from the linear mixed-effects models could not be provided. In order to quantify the difference in either amplitude or occurrence rate between a tested location and the orbitofrontal reference, population level predictions were computed for each location. An inverse Box-Cox transformation was then applied to these estimates to obtain values in either microvolts or milliseconds in order to compute a relative percentage to the reference level.

To test whether KCs co-occur in multiple channels, we compared the number of KC events observed in exactly one channel, versus more than one channel, to what those numbers would be by chance. Fisher’s exact test was performed in R to determine whether the numbers significantly differed at α = 0.05. The number of KCs that would be expected in one channel or multiple channels by chance was estimated by randomizing the latencies between two consecutive KCs within each channel. This provided a new list of KC peaks for each channel. This randomization was performed 1000 times. These randomized latency differences were converted into latencies by adding in the latency of the first detected KC for each channel. Additionally, the latency of the first detected KC was added back to keep the number of latencies consistent. The KCs were then grouped for each of these 1000 s simulations in the same manner as for the actual data (i.e., within 200 ms time windows) to create the distribution of expected single and multichannel KC events under the null hypothesis.

Using the same method for subjects with more than two channels, we tested co-occurrence for KCs observed in three or more channels, four or more channels, and five or more channels. Five or more KCs had to be observed for the category to be tested. Bonferroni correction for multiple comparisons was applied to all test results.

To test whether KCs occur in a sequential order across the cortex, a multinomial test was performed in R for KC events observed in two, three, four, or five channels. The channel sequence of each KC event was determined by ordering channels by their KC peak latency. For a given set of channels, the multinomial test compares the frequencies of observed sequences to all possible sequences containing the same channels. Bonferroni correction was applied separately to each category of channels over all subjects.

Because each KC was verified visually, and for each channel, KCs were in all cases validated by decreased high gamma power, one may be confident in the locations where they were found to be locally generated and other quantified characteristics. However, using such strict criteria could lead to an underestimation of the extent of cortical involvement in a given KC event. This possibility was avoided by using an automatic procedure to look for KC-like activity in the channels where KCs were not manually marked, at times when a KC was manually marked in at least one channel. Each channel was bandpassed with a zero phase-shift filter from 0.2 to 5 Hz before the detection of KC-like activity. For each channel, a KC template was created by averaging over the manual KC detections from −350 to +650 ms around the peak. A sliding inner product was then performed between this template and a 1.2 s window when a manual KC occurred in at least one channel. To prevent edge effects, this window was set from −450 to +750 ms around the manually marked KC peak at time zero; if more than one channel was manually marked, then the average of the first and last peak latency was used as time zero. The maximum value of the sliding inner product within the 200 ms window where the template and test data overlap was compared with a predetermined threshold value. If this value was greater, the channel was considered to contain KC-like activity. However, if the time when the sliding inner product was greatest was at the edge of the 200 ms window (and the amplitude continued to get larger beyond that window), then the activity was not counted as KC like.

The threshold value for KC-like activity was determined for each channel by calculating the sliding inner product between the KC template and randomly chosen epochs of data that did not contain KCs or slow oscillations in any channel. The number of such epochs chosen was 110% the number of KC events for each night. As above, the maximum value of the 200 ms window where the template and each epoch overlapped was stored in a vector. The threshold was the 99th percentile of that vector. Thus, the KC-like activity chosen by the template can be considered to be more similar than random activity to the template constructed from the manually chosen KCs at *p* < 0.01. For 2 nights in two different subjects, it was not possible to obtain 110% the number of KC events. In these cases, ∼350 periods for each channel were used.

To quantify the characteristic differences between manually detected and template-detected KCs, the KC peak amplitudes, and the signal variance from −1.4 to −0.4 s prior to the KC peak, were calculated for each channel for the null times, manual KCs, and template-detected KCs. The *z*-scores for the peak amplitude and variance on each channel for the manually detected and template-detected KCs were calculated with respect to the null values. A paired *t* test was calculated between the *z*-scored manual KC amplitudes averaged over all channels and the *z*-scored template-detected KC amplitudes averaged over all channels. Another paired *t* test was calculated comparing the pre-KC period variance between average *z*-scored manually detected and template-detected KCs.

A new list of KC events, including KC-like activity in addition to the manually marked KCs was computed. To test whether there is a consistent temporal relationship between manually detected and template-detected KCs, the difference between the average peak latency of all manual KCs and the average of all template KCs was calculated for each KC event for a representative night over all subjects.

The number of channels participating in KCs and the regional distribution of KCs was compared between the manual-only KCs and the manual plus template-detected KCs. For 1 representative night for each subject, the number of KCs occurring on each channel was normalized to the largest number of KCs occurring on a channel. This normalization was performed separately for the manual-only detections and the manual plus detections surviving the 99th percentile threshold. A multinomial test was repeated for these KCs to test whether the inclusion of KC-like activity revealed any meaningful sequential order of two channels across the cortex. Bonferroni correction was applied to all tests.

To further test for a significant relationship between KC latency and anterior-to-posterior distance in the cortex, all pairs of anterior and posterior channels were chosen for each subject. Pairs were in all cases from different electrodes and could maximally have one contact gap in the medial-lateral direction. These three-dimensional electrode coordinates were projected onto the sagittal plane. The distance between these coordinates for each channel pair was plotted versus the average KC latency difference between each pair. Additionally, the channel distance was plotted for each individual KC latency difference. Significant distance versus delay relationships were analyzed in a linear mixed-effects model (Pinheiro et al., 2015), where the patient was introduced as a random effect to account for interindividual variability. Marginal *r* ^2^ values were calculated as presented in the study by Nakagawa and Schielzeth (2013).

## Results

### Cortical areas generating KCs

KCs were analyzed from nine patients undergoing observation for intractable epilepsy. A total of 55 bipolar SEEG contacts were included for analysis. Contacts sampled all lobes but showed the greatest concentration in subcallosal, prefrontal (anterior and posterior), and temporo-parieto-occipital junction regions (Fig. 1*A*). Bipolar contact pairs spanned the gray matter, with one contact in the white matter and the other in the CSF, providing a very focal measure (Fig. 1*B*).

**Figure 2.**
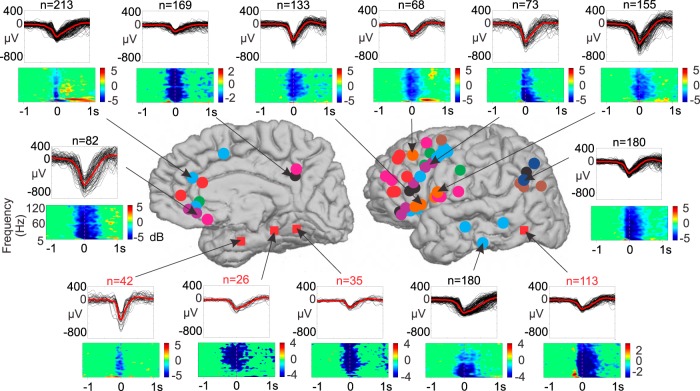
Example KC localizations, waveforms, and down state confirmations. KCs are plotted from representative sites across the cortical areas sampled. Individual KCs for each bipolar location are plotted in black with the average KC overlaid in red. The number of individual KCs plotted is listed above the waveforms. Below the KC waveforms, time frequency plots from 5 to 120 Hz with a −1.5 to −0.5 s baseline relative to the most negative peak of the KC at time zero indicate a significant drop (*p* < 0.01, uncorrected) in high gamma power at the time of the KC. Arrows indicate the bipolar locations where the plotted waveforms and time–frequency plots were recorded. Circles indicate the subject color-coded bipolar channel locations, as in Figure 1. The four additional red squares indicate sites where KCs were located, but not included for additional analysis because they were part of an epileptic region or potentially in entorhinal cortex.

KCs were manually marked, independently on each bipolar channel. A total of 13,821 KCs were manually marked over all channels for all subjects. For each channel, KCs were verified as down states by a significant drop in high gamma power at the time of the KC (Fig. 2). Locally generated KCs were demonstrated in all sampled regions, including subcallosal, orbital, cingulate (anterior and posterior), frontal (superior, middle and inferior, pars opercularis, triangularis, and orbitalis), supramarginal, angular, annectant, temporal (inferior and middle) gyri, the insula and frontal operculum, and the central and anterior occipital sulci (Fig. 2).

In addition to the 55 bipolar SEEG contacts included for further analysis, KCs were also identified in four bipolar SEEG contacts where it has previously been reported that KCs are not found (Wennberg, 2010). These sites were in anteroventral medial temporal (possibly entorhinal), posteroventral medial temporal, and lateral ventral temporal areas. These four sites were not included in further analyses because they either occurred in potentially non-isocortex (entorhinal cortex) or were in locations implicated in the patient’s epilepsy; therefore, further verification is needed for these sites. As with the other pairs, these sites recorded locally generated KCs, as verified by a significant drop in high gamma power (Fig. 2, sites marked by red squares).

### KCs measured in bipolar versus common average reference montage

**Figure 3.**
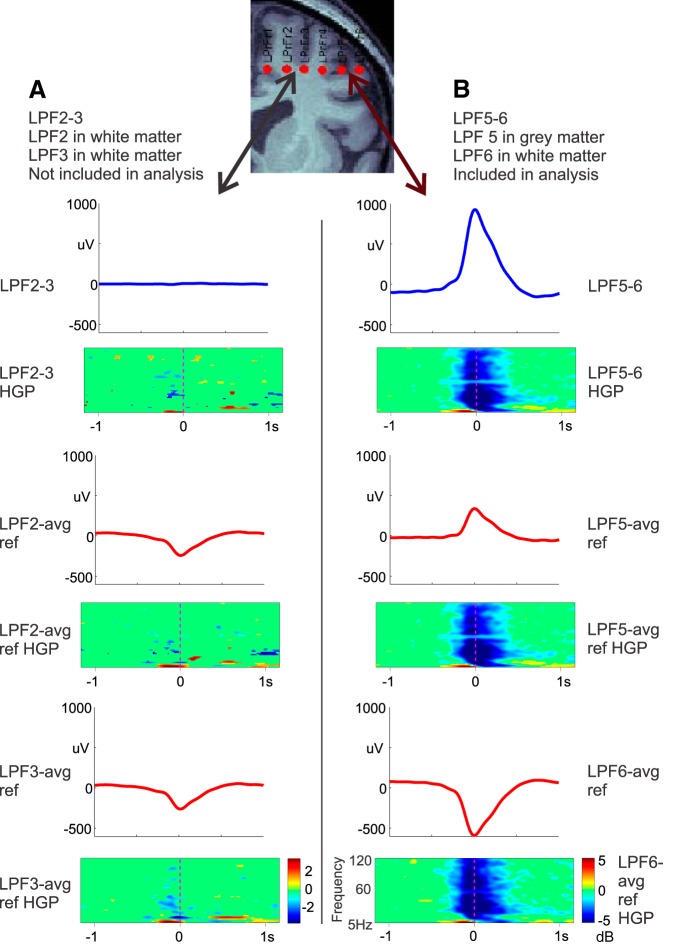
Bipolar versus common average reference recordings. Bipolar KC recordings (blue waveforms) were compared with monopolar KC recordings (red waveforms) referenced to a common average reference (T3, T5, F3, C3, P3, O1, F4, C4, P4, O2, T4, and T6) as in Wennberg (2010). Two pairs of adjacent contacts located on the same electrode of subject 4 were examined. ***A***, Bipolar (blue) and referential (red) recordings from two adjacent contacts, both located in the white matter, as confirmed by MRI. Bipolar recordings show no KCs, but referential recordings do, despite the fact that no decrease in high gamma power is present in the time–frequency plots below each waveform. Thus, referential recordings show KCs even when they are not locally generated. ***B***, Bipolar (blue) and referential (red) recordings from two adjacent contacts that span the gray matter, as confirmed by MRI. Both bipolar and referential recordings show KCs, together with decreased high gamma power. All recordings were averaged with respect to the peaks of KCs identified in the bipolar recordings in ***B***.

Previously, KC generation was examined using SEEG, with each individual electrode contact referenced to a common average of scalp electrodes (Wennberg, 2010). To compare this method to the method of transcortical bipolar subtraction used in the analyses presented here, KCs were examined in two adjacent white matter contacts, as well as two adjacent contacts spanning the gray matter, in both the common average reference and bipolar montage (Fig. 3). All four contacts were located on the same electrode.

Using the KCs identified on the bipolar channel spanning the gray matter, the two white matter contacts referenced to the common scalp average show clear KCs (Fig. 3*A*, red waveforms). However, when the two white matter channels are viewed in a bipolar montage (blue waveform), the signal is flat. This finding implies that the common average reference signal in this case is due to volume conduction rather than local generation. This interpretation is consistent with the absence of a drop in high gamma power in any bipolar or referential recording from the white matter contacts.

Conversely, the transcortical bipolar contact along the same electrode used in this study spans the gray matter, with one contact on the white matter side of a cortical patch, and the other on the CSF side. In this case, both channels referenced to the common scalp average show KCs (Fig. 3*B*, red waveforms), but so does the bipolar subtraction between these two channels (Fig. 3*B*, blue waveform). Additionally, in all three cases, there is a drop in high gamma power to verify that what is being recorded is a down state. A common average reference SEEG can, therefore, accurately record KCs, but it also risks recording activity that is not locally present, which is not the case for bipolar SEEG.

### KC amplitude and occurrence rate varies across cortical locations

**Figure 4.**
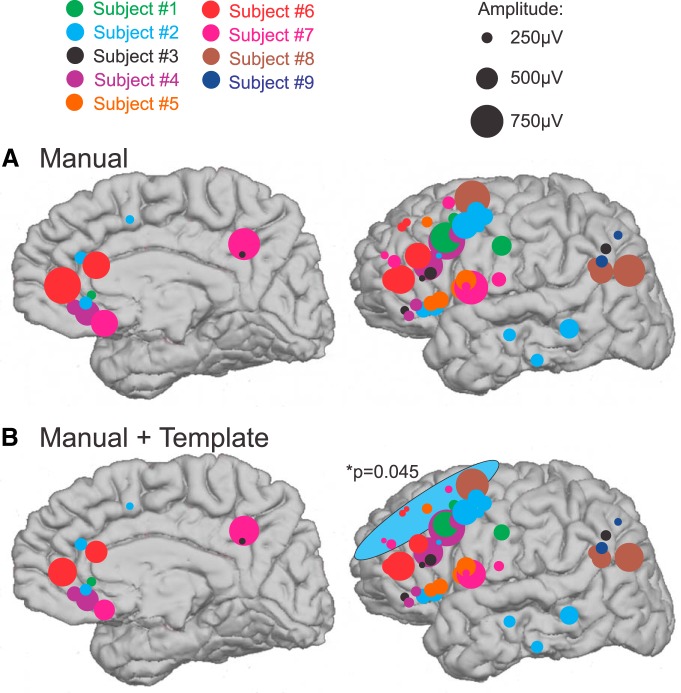
KC amplitudes vary across the cortex. ***A***, The average amplitude of manual KCs at each channel, color coded by subject, is highly variable across the cortex. However, no significant effects are found when a linear mixed-effects model is applied. ***B***, The addition of KC-like activity shows a similar pattern, but with the increased sample, the same linear mixed-effects model now finds that the anterior prefrontal channels highlighted by the blue circle are ∼30% smaller than the orbitofrontal reference (*p* = 0.045). Black circles in the top right indicate the amplitude scale represented by the size of each circle.

In Figure 4*A*, average KC amplitudes for each channel are color coded by subject. The mean amplitudes for subjects 1 through 9 were as follows (in μV): 416, 329, 209, 489, 385, 513, 420, 624, and 230. KCs with the largest amplitude were recorded in posterior prefrontal and anterior cingulate/subcallosal areas, whereas small KCs were recorded in anterior prefrontal and orbital areas, and moderate amplitude KCs were recorded in ventrolateral temporal areas (Figs. 2, 4*A*). Large KCs were also recorded in supramarginal, posterior cingulate, and anterior occipital areas, although sampling was limited. It should be appreciated that low amplitudes could reflect individual variation between patients, in exact electrode placement with respect to the cortex, or in variable spacing between contacts, rather than a characteristic of a particular cortical area.

In order to account for these patient and channel variabilities, a linear mixed-effects model of the amplitudes was performed with location as a fixed effect, patient as a random effect, and channel as a random nested effect. Bipolar channels were grouped into 10 locations by anatomic location, as pictured in Figure 1. The results of the model revealed that there were no significant amplitude differences between areas, although the anterior prefrontal cortex was close to being significantly smaller (*p* = 0.089), despite the common observation that the largest scalp KCs are recorded above this area. These issues are further examined below.

**Figure 5.**
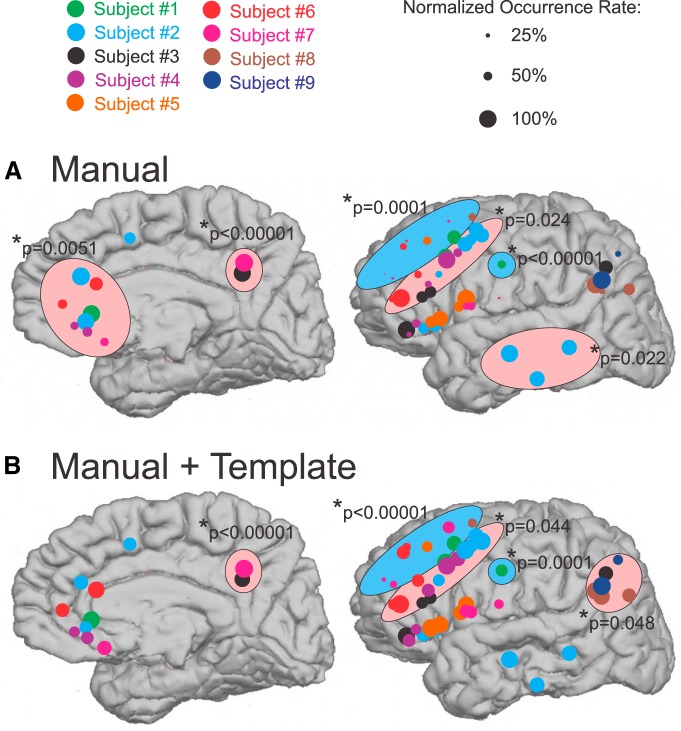
KC occurrence rates vary across the cortex. ***A***, Manually marked KCs color coded for each subject. The size of the circle indicates the KC occurrence rate at each location, normalized to the maximum KC occurrence rate seen on any channel for that subject. Black circles in the top right indicate this normalized occurrence rate scale. Locations identified by a linear mixed-effects model to have a significantly higher occurrence rate than the orbitofrontal reference are highlighted by pink circles. Locations identified as occurring significantly less frequently are highlighted by blue circles. ***B***, KC-like activity in addition to manually marked KCs. The locations showing a lower occurrence rate are the same as in ***A***. The locations showing a higher occurrence rate differ with the addition of the template-detected KCs.

In Figure 5*A*, the occurrence rate in each bipolar pair was normalized to the pair with the highest frequency in that subject, resulting in less striking differences between cortical locations. As a measure of occurrence rate, the inter-KC intervals were calculated for each channel. A linear mixed-effects model of these intervals was calculated with location as a fixed effect and patient as a random effect to test for areas with significantly different occurrence rates. Compared with the orbitofrontal reference, KCs occurred 15% more often in the anterior cingulate (*p* = 0.0051), 48% more often in posterior cingulate (*p* < 0.00001), 11% more often in posterior prefrontal cortex (*p* = 0.024), and 14% more often in ventrolateral temporal regions (*p* = 0.022); these groups are highlighted in pink in Figure 5*A*. Again, contrary to inferences from scalp EEG findings, KCs occurred 30% less often in the anterior prefrontal cortex (*p* = 0.0001) compared with the orbitofrontal location. KCs also occurred two times less frequently in the central sulcus (*p* < 0.00001); these two locations are highlighted in blue in Figure 5*A*.

### KCs co-occur in channels across the cortex

In order to determine whether KCs co-occur across the cortex, the KCs that were marked independently on each channel were regrouped. Starting with the first KC latency, any KCs occurring in other channels within the next 200 ms were grouped as being part of the same KC event. As the time delay of potential KC propagation across the cortex is unknown, this 200 ms time window was defined for grouping a KC event because it is the approximate time delay reported for the slow oscillation from anterior to posterior scalp EEG recording sites (Massimini et al., 2004). Each KC event, therefore, would minimally contain one channel and could maximally contain the number of channels analyzed for an individual subject.

To examine whether KC peaks in different channels participating in a KC spanned the entire 200 ms window, the time between the first and last peak in each KC containing more than one channel was calculated. The mean and SD of these maximum peak delays was calculated for each night for each subject. All subjects showed a mean peak difference of <100 ms (Table 2). The single exception was 1 night for subject 9, where only one KC involved both channels; as a result, the mean reflects only one calculation. Therefore, even though a 200 ms window was chosen to include KCs potentially traveling across the cortex, KCs occurred in almost all cases in less than half this time.

**Table 2.** Mean and SD of maximum KC peak differences between manually chosen KCs for each night for each subject

Subject	Channels (*n*)	Mean of maximum KC peak differences (ms)	SD (ms)
1	4	61.1	49.5
2	13	99	59.6
3	5	86.3	59.5
4	7	66.3	54.1
5			
N1	5	24.8	23.8
N2	5	27.8	26.4
N3	5	32.2	27.7
6			
N1	7	71.3	48.5
N2	7	66.4	45.6
N3	7	81.2	52.8
N4	7	60.3	54
7			
N1	8	68.6	59.1
N2	8	63.2	57.2
N3	8	73.4	61.2
N4	8	77.7	63.1
N5	8	70.7	51.1
N6	8	74.9	59.7
8			
N1	4	49.8	53.4
N2	4	79.2	42
N3	4	67.5	59.7
9			
N1	2	95.2	59.4
N2	2	115.2	0
N3	2	52.2	39
N4	2	78.6	83

**Figure 6.**
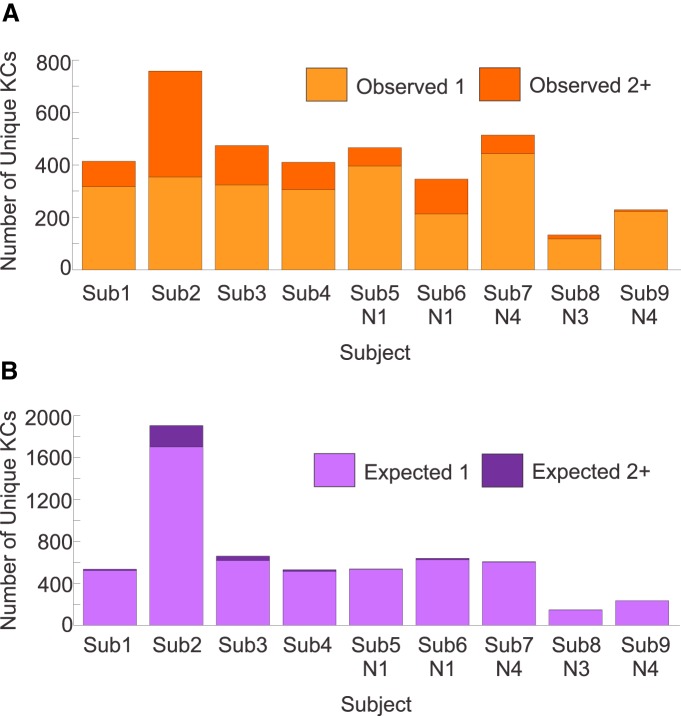
Observed manual KCs versus those expected under the null hypothesis of independent occurrence, for representative nights for each subject. ***A***, KC events observed for each subject were classified into two groups: KCs with one participating channel (light orange); and KCs with two or more participating channels (dark orange). ***B***, KC events derived from simulating an expected distribution for each subject were classified into the same two groups: one channel (light purple); and two or more channels (dark purple). For all subjects, there are more KCs that occur only in one channel expected under the null hypothesis than were actually observed. These distributions are tested statistically using a Fisher’s exact test, and these results are listed in Table 3.

To test whether these observed KC co-occurrences were significant, we simulated a set of expected KC data under the null hypothesis that the KC times are unrelated in different channels. This expected distribution was created by randomizing the inter-KC intervals within channels of the observed KCs and grouping the latencies over channels within 200 ms windows, as was done with the observed KCs. We examined observed and expected KCs occurring in one channel versus two or more channels for each subject (Fig. 6). In the observed KCs, there are KCs that occur only in one channel (Fig. 6*A*, light orange). However, there are many more KCs that occur only in one channel for the null distribution (Fig. 6*B*, light purple). Conversely, there is a larger number of KCs involving two or more channels for the observed data (Fig. 6*A*, dark orange) than under the null hypothesis (Fig. 6*B*, dark purple). To statistically verify this observation, a Fisher’s exact test was performed, comparing the number of observed KCs to the null distribution in one channel versus two or more channels. For 20 nights over eight subjects, this was highly significant (*p* < 0.05, Bonferroni corrected; Table 3). For 4 nights over two subjects (3 of 4 nights for one subject and 1 of 3 nights for the other subject), it was not. Due to the variable number of channels for each subject, the “one channel” versus “two or more channels” comparison was the only comparison testable for all nine subjects. In eight subjects, it was possible to repeat this test using expected and observed data for one channel and three or more channels; for seven of these subjects at least 1 night was significant at *p* < 0.05 (Bonferroni corrected), while the night of the eighth subject was not significant. For six subjects, it was possible to test up to four or more channels; in this case, we find all nights for five subjects to be significant at *p* < 0.05 (Bonferroni corrected) and not in 1 night in one subject. This analysis demonstrates that KCs co-occur across the cortex.

**Table 3.** Fisher’s exact test for co-occurrence for one channel and two or more channels; three or more channels; four or more channels; and five or more channels for all nights for all subjects

Subject	1 Channel and ≥2 channels	1 Channel and ≥3 channels	1 Channel and ≥4 channels	1 Channel and ≥5 channels
1	**< 2.2E-16**	**9.49E-12**	**0.000445**	
2	**< 2.2E-16**	**< 2.2E-16**	**< 2.2E-16**	**< 2.2E-16**
3	**< 2.2E-16**	**< 2.2E-16**	**6.24E-09**	0.00493
4	**< 2.2E-16**	**< 2.2E-16**	**5.38E-08**	
5				
N1	**< 2.2E-16**			
N2	**5.40E-14**			
N3	**2.96E-13**	0.0135		
6				
N1	**< 2.2E-16**	**< 2.2E-16**	**< 2.2E-16**	**1.10E-15**
N2	**< 2.2E-16**	**< 2.2E-16**	**< 2.2E-16**	**< 2.2E-16**
N3	**< 2.2E-16**	**< 2.2E-16**	**< 2.2E-16**	**1.25E-15**
N4	**3.92E-10**	0.0125		
7				
N1	**< 2.2E-16**	**2.33E-06**		
N2	**< 2.2E-16**	**1.20E-05**		
N3	**< 2.2E-16**	**1.61E-05**	0.0140	
N4	**< 2.2E-16**	**2.00E-08**		
N5	**9.50E-06**			
N6	**8.09E-08**			
8				
N1	**< 2.2E-16**	**0.000355**		
N2	0.00227			
N3	**0.000107**			
9				
N1	0.215			
N2	0.497			
N3	**0.000312**			
N4	0.0140			

Bonferroni correction at *p* < 0.05 was applied across all tests. Values in bold are significant after this Bonferroni correction.

### KCs do not occur in particular sequential order across the cortex

As examined above, channels participating in a KC event exhibit a tighter time window than the 200 ms used to group KC events. However, this specific limit was imposed to investigate propagation sequences: according to Massimini et al. (2004), it takes ∼200 ms for a slow wave to travel from anterior to posterior cortex. As stated previously, because the potential propagation time of KCs is unknown, the time delay of the SO was used as a maximum time window. To test the hypothesis that KCs occur in a sequential order across the cortex, we performed a multinomial test on the sequential order of channels participating in the observed KCs. Using the KC peak latencies, we recorded for a pair of channels the number of times the KC peak occurred in channel A before channel B and the number of times the KC peak occurred in channel B before channel A. In this example of two channels, the multinomial test determined when one sequential order occurred significantly more often than the other sequential order. Of 341 channel pairs tested over a representative night for each of the nine subjects, only 20 pairs from two subjects (subjects 2 and 6) were statistically significant at *p* < 0.05 after Bonferroni correction. These results indicate that when two channels repeatedly record KCs within a 200 ms time window, only 6% of these 341 channel pairs show a significantly consistent latency order. Conversely, 94% of channel pairs show no systematic latency even though they occur repeatedly within the same transcortical KCs.

**Figure 7.**
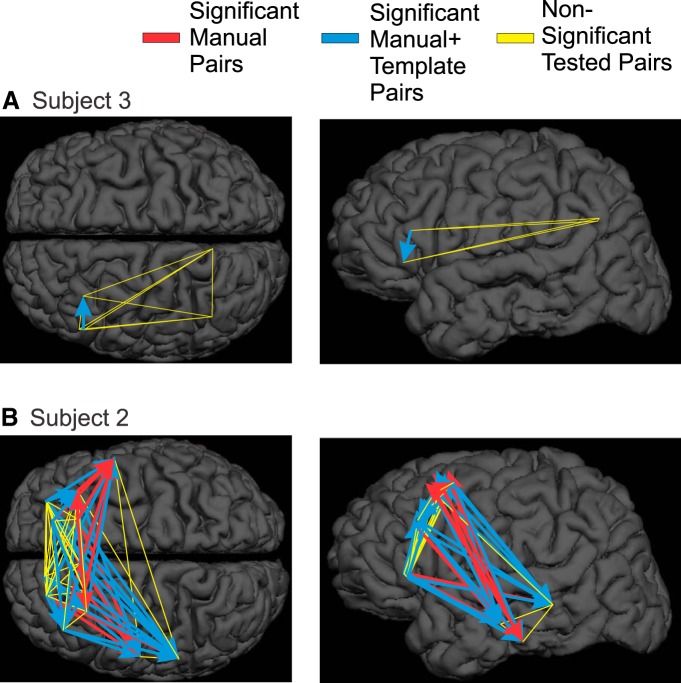
Lack of systematic sequential order in KCs. Sequence pairs that are significant with manually marked KCs are plotted with red arrows, while sequence pairs that are significant only after the addition of KC-like activity are plotted with blue arrows. Sequence pairs tested but not significant are plotted in yellow. ***A***, Subject 3 has electrodes in frontal and parietal cortices, but only shows significant sequences within the frontal lobe, and only after the addition of KC-like activity. ***B***, Subject 2 shows a large number of significant sequence pairs (16) in manually marked KCs, the majority of which lead to the temporal lobe (11). This pattern is strengthened with the addition of KC-like activity (18 of 29 additional significant pairs). A variety of other significant sequences are seen within the frontal lobe.

If KCs significantly occurred in a sequential order from anterior to posterior cortex, we would expect to find ordered sequences of two or more channels, beginning in anterior cortical regions and finishing in posterior cortical regions. Thus, we examined individually all of the electrode pairs to determine whether they were systematically engaged in an anterior-to-posterior direction. We found that for subject 2, 11 of the 16 significant pairs started in various parts of cortex and ended in the temporal lobe. Three other pairs were situated along the same electrode, one was a pair of mirrored locations across the hemispheres, and the last was a pair with a slight posterior-to-anterior direction (Fig. 7*B*, red arrows). Of the four significant pairs found for subject 6, there was evidence of anterior-to-posterior propagation for three pairs, all lying entirely within prefrontal cortex. One of these three pairs was along the cingulate gyrus. One other pair was located along the same electrode, with no anterior–posterior separation.

We further tested three-, four-, and five-channel sequences, whenever possible given the number of channels and the rate of multichannel KC occurrence for each subject. Of 222 triplets tested, 5 from subject 6 were statistically significant at *p* < 0.05 after Bonferroni correction. As with the pair results for this subject, these triplets showed anterior-to-posterior propagation only within the prefrontal cortex, as well as patterns that bounced back and forth or looped around within the frontal cortex. There were no significant sequences involving four or five channels.

### Automatic template-based detection of KC-like activity

**Figure 8.**
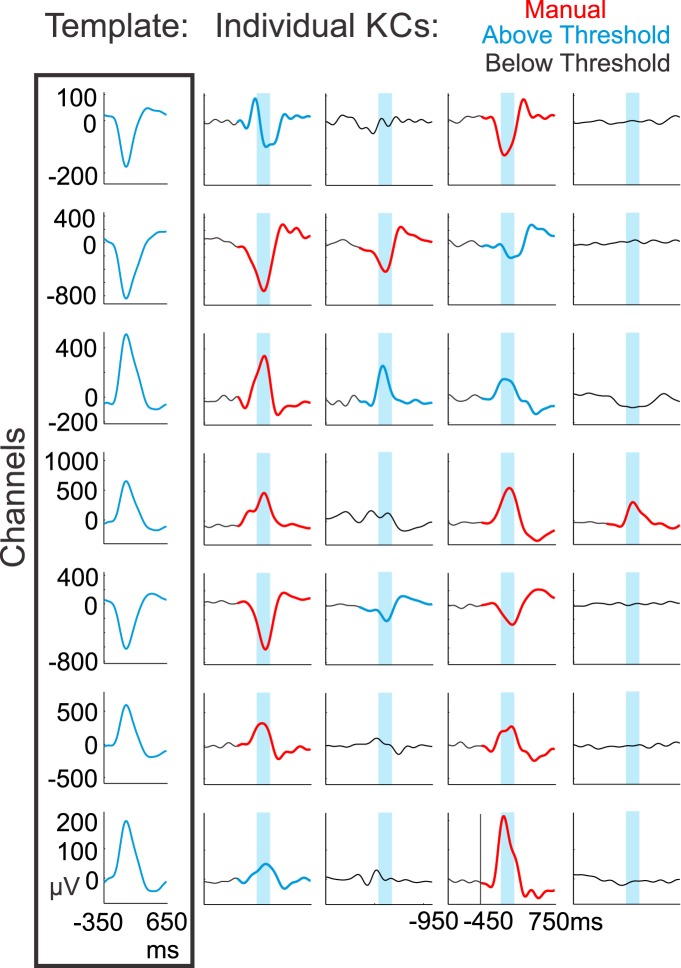
Template application to detect KC-like activity. Channel-specific templates were created for each subject by averaging from −350 to +650 ms on the most negative peak at time zero over the manually detected KCs in each channel (box at left). The channel-specific templates were then applied when a manually marked KC occurred in at least one channel. The templates were applied from −450 to +750 ms, with time zero representing the average time between the first and last manually chosen channel peaks within a KC. Four such examples from subject 6 are plotted vertically. Manually marked KCs are plotted in red. The maximum value of the sliding inner product between the signal and the template was taken over the blue highlighted window. If the value was above the 99th percentile of the null distribution for that channel and corresponded to the largest (or smallest) peak over the entire 1200 ms window, the KC was recorded as KC-like activity (KCs in blue), and if it was below the threshold, it was not (signal in black).

The above analyses were performed on the manually marked KCs. The strict criteria used to select these phenomena, most notably that there was a flat baseline (i.e., no preceding oscillation) prior to the KC, may have prevented KC-like phenomena from being detected, and thus result in an underestimation of the degree of KC co-occurrence. In order to detect KC-like events, a template was created for each channel and applied at the times of manual KCs occurring in at least one channel (Fig. 8). A threshold was established as the 99th percentile of periods when no KC was visually present. Events that exceeded the template threshold were included as KC-like events (see Materials and Methods). Application of these individualized channel templates identified 13,383 KC-like events—a 96.8% increase in detected KCs—and 98.7% of the 13,821 manually marked KCs.

**Figure 9.**
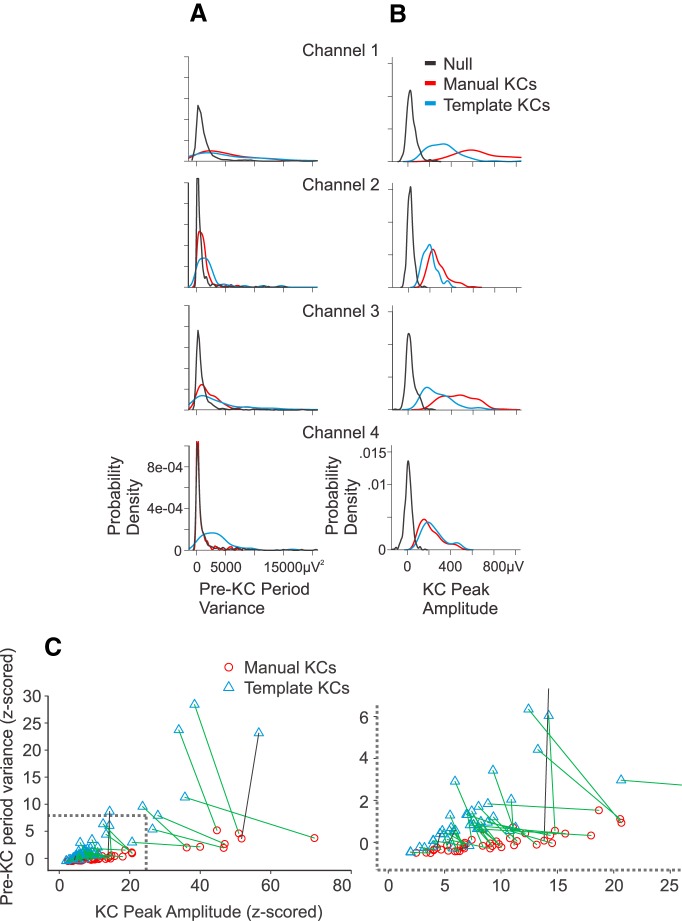
Manually detected KCs have smaller pre-KC period variance and larger amplitude than template-detected KCs. ***A***, The probability density of variance in the pre-KC period (−1.4 to −0.4 s prior to peak) is plotted for the null times (black), manually detected KCs (red), and template-detected KCs (blue) for each of subject 1’s four channels. ***B***, The KC peak amplitude probability density of null times (black), manually detected KCs (red), and template-detected KCs (blue) are plotted for the same four channels as in ***A***. ***C***, For each channel, the manually detected (red circles) and template-detected (blue triangles) *z*-scores were calculated for the KC peak amplitude and the pre-KC period variance referenced to the null values. A line connects the manually detected and template-detected values for each channel. A green line indicates that the amplitude of template-detected KCs is smaller than that of manually detected KCs in that channel; a black line indicates the opposite relationship. On the right is an expansion of the lower left corner, which is outlined by a gray box.

Compared to the manually detected KCs, template-detected KCs were smaller in amplitude (manual = 420 ± 219 µV vs template = 326 ± 183 µV) and had more variance in the pre-KC period (manual KCs = 1945 ± 1604 vs template-detected KCs = 7388 ± 10,708). The variance in the preactivity period (Fig. 9*A*) and the KC peak amplitude (Fig. 9*B*) for each of subject 1’s four channels are plotted for the null times (Fig. 9*B*, black distribution), manual KCs (Fig. 9*B*, red distribution), and template-detected KCs (Fig. 9*B*, blue distribution). In some cases, the template-detected KCs were smaller in amplitude than the manual KCs, without much distinction in the variance (channel 1), while in other cases, the opposite occurred: the variance was larger for template-detected KCs than for manual KCs, without a large distinction in amplitude (channel 4). In most cases, it was a combination of variance and amplitude that distinguishes the manually detected and template-detected KCs (channels 2 and 3).

In order to evaluate for all channels the contributions of KC peak amplitude and pre-KC period variance in distinguishing manual from template-detected KCs, *z*-scores were calculated for the KC peak amplitude and pre-KC period variance for each channel with respect to the null values (Fig. 9*C*, right, which is an expansion of the lower left corner outlined in gray). In general, as above, the manual KCs (Fig. 9*C*, red circles) are larger in amplitude (i.e. shifted to the right on the *x*-axis) than the template-detected KCs (Fig. 9*C*, blue triangles). The manually detected and template-detected values for each channel are connected by a line. This line is green if the manual KCs were larger in amplitude than the template-detected KCs; however, this line is black in the rare cases where the template-detected KCs are larger in amplitude than the manual KCs. Furthermore, the pre-KC period variance is generally larger (i.e. shifted up on the *y*-axis) for the template-detected KCs compared to the manual KCs within each channel. To test these observations, a paired *t* test between the manually detected and template-detected KC *z*-transformed amplitudes over all channels was significant (*p* = 0.000065), as was a paired *t* test between the *z*-transformed variance in the pre-KC period for manually detected and template-detected KCs over all channels (*p* = 0.00027). While there are clear overall distinctions in amplitude and pre-KC period variance between template-detected and manually marked KCs, they appear to form a continuum on a channel-by-channel basis, and thus both manually detected and template-detected KCs should be considered together when examining the true extent of cortical involvement at the time of KCs.

We further examined whether a directionally consistent temporal lag existed between manually detected and template-detected KCs by subtracting the average of the template KC peaks from the average of the manual KC peaks within each KC event. The mean lag was −1.25 ms with an SD of 40 ms, and the normality of the distribution was determined visually; therefore, we concluded that no such temporal relationship existed between the manually detected and template-detected KCs.

### Including KC-like activity increases the multichannel participation of KCs

**Figure 10.**
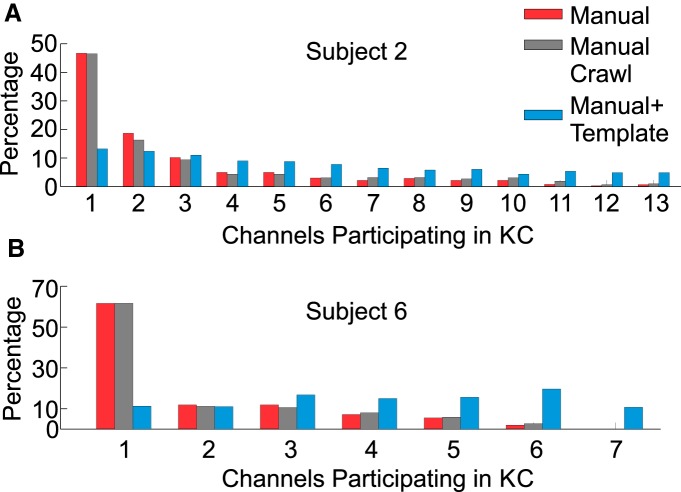
Including KC-like activity increases multichannel KC participation. The addition of KC-like activity (blue columns) to the manually marked KCs (red columns) increased the number of channels that were participating in KCs for all subjects. ***A***, In subjects where a small number of manually marked KCs included the participation of all channels, the addition of KC-like activity increased the percentage of such KCs. ***B***, Subjects who did not show maximum channel participation with manually marked KCs displayed KCs that included all channels after the addition of KC-like activity. In subjects 2 and 6 (pictured), and subject 3 (not pictured), the classification of KCs using a 200 ms crawl window slightly shifted the distribution of manual multichannel participation to the right (gray columns), but not to the extent of the addition of KC-like activity (blue columns).

Combining the number of manually marked KCs with the template KCs resulted in 27,204 total KCs over all subjects. We first examined how the addition of template KCs increased multichannel participation. In subjects where all channels participated in a subset of manually marked KCs, the percentage of such KCs increased with the addition of template-detected KCs (Fig. 10*A*). Similarly, in subjects where only a subset of channels participated in manually marked KCs, the addition of template KCs resulted in KCs where all possible channels participated (Fig. 10*B*). Manually marked KCs already showed significant co-occurrence across the cortex; the addition of KC-like activity indicates that KCs co-occur even more broadly across the cortex.

In Subjects 1, 2, 3, and 9, all channels participated in a subset of manually marked KCs. For subject 1, the percentage of KCs involving all four channels increased from about 2% in manual KCs to 9% after addition of the template KCs. For subject 2 (Fig. 10*A*), all 13 channels participated in only 0.7% of manual KCs, which increased to ∼5% of all KCs with the addition of KC-like activity. If ≥10 channels are considered as a group for subject 2, then the percentage of total KCs increased from ∼4% in the manual case to 20% in the case of manual plus template-detected KCs. For subject 3, the increase in KCs involving all channels was slight, from 1% to 2.5%, while for subject 9 this increase was from ∼3% to ∼28%. While this latter result indicates the largest increase, there are only two channels for subject 9.

In subjects 4, 5, 6, 7, and 8, KCs with all channels participating were not found until template-detected KCs were included. In subject 4, only five of seven channels maximally participate in manually marked KCs, and these represent only 0.2% of KCs. With the addition of template-detected KCs, KCs may now involve five, six, or seven channels, accounting for >12% of all of the KCs for subject 4. For subject 5, manual KCs only maximally involve three of five possible channels, representing 0.6% of KCs. After template detections, >38% of the subject’s KCs involve three, four, or five channels. For subject 6 (Fig. 10*B*), only 2% of manually marked KCs involve six of seven channels. After template application, this increases to almost 20%, and the number of KCs involving all seven channels is just under 11%. In subject 7, a maximum of only five of eight possible channels participate in manual KCs, and 86% of KCs only involve one channel. After template detection, KCs involving six, seven, and eight channels collectively account for ∼7% of KCs. For subject 8, only three of four channels maximally participate in the manual KCs, but after including KC-like activity, ∼6% of KCs include all four channels.

A 200 ms detection window on manually marked KCs was chosen based on the differences in KC peak latencies reported at the scalp for SOs (Massimini et al., 2004). However, if this window was artificially small, then we may have underestimated the true extent of KC co-occurrence. In order to test for this possibility, we recalculated co-occurrence after grouping KCs using a 200 ms “crawl” window (Fig. 10). In the analyses described above, we start at the first KC peak and search within the following 200 ms window for any other manually marked KC peaks in other channels. In the crawl analysis, we follow the same procedure, but shift the 200 ms window to follow the second KC peak. The window continues to shift until there are no further detections within the 200 ms window. The recalculated co-occurrence histograms showed minimal changes using this procedure. Only three of the nine subjects (subjects 2, 3, and 6) showed an increase of more than one KC with the participation of all channels. Even in these subjects, the effects were minimal, as seen by comparing the red and gray bars in Figure 10. In contrast, adding template-detected KCs had a substantial effect on co-occurrence, as seen by comparing the red and blue bars in Figure 10.

In summary, including only the manually marked KCs, most KCs (76%, averaged across all subjects) occurred in a single location, and rarely (1%) in all locations. However, if template-detected KCs were included, only 15% occurred in a single location, and 27% occurred in all locations.

### After including KC-like activity, KCs are smallest in anterior prefrontal regions

In Figure 4*B*, average KC amplitudes, including both manually detected and template-detected KCs, are shown for each channel (color coded by subject). For comparison, the same amplitude data for manual KCs only are plotted in Figure 4*A*. The addition of the template KCs generally decreases the average KC amplitude at each channel, as seen by a reduction in circle size between Figure 4*A* and Figure 4*B*, and as shown to be significantly different in Figure 9*C*.

In order to compare the amplitude of KCs in different areas, a linear mixed-effects model of the amplitudes was again performed. The anterior prefrontal channels were found to be ∼30% smaller than the orbitofrontal reference channels (*p* = 0.045; Fig. 4*B*, blue circle). Again, this finding is in contrast to what would be predicted by scalp EEG.

### Adding KC-like activity has minor effects on the pattern of KC occurrence rates across cortical locations

We next examined how the addition of KC-like activity influences the spatial distribution of KC occurrence rates across the cortex. Again, in Figure 5*B*, the average KC occurrence rate for each channel is color coded by subject, including both manually detected and template-detected KCs, for comparison with the same occurrence rate data plotted for manual KCs only in Figure 5*A*. For each subject, the occurrence rate of KCs in each channel is normalized to the channel with the highest KC occurrence rate.

Similarly, in order to test whether the KC occurrence rate varies across locations after including template-detected KCs, a linear mixed-effects model was again applied to the inter-KC intervals for each channel. Compared to the orbitofrontal reference, KCs still occur significantly more frequently in posterior cingulate (26% more; *p* < 0.00001) and posterior prefrontal (6% more; *p* = 0.044). They no longer occur more frequently in anterior cingulate or ventrolateral temporal cortex, but KCs now occur more frequently at the temporo-parieto-occipital junction (15% more; *p* = 0.048). These groups are highlighted in pink in Figure 5*B*. As was the case with the manual KCs, the anterior prefrontal KCs occur 26% less frequently (*p* < 0.00001) and the central sulcus KCs occur 48% less frequently (*p* = 0.0001) than those in the orbitofrontal channels and are highlighted in blue in Figure 5*B*.

### Adding KC-like activity does not reveal any systematic sequential order across the cortex

We tested whether using only manually marked KCs undersampled KC activity and therefore, resulted in the apparent lack of any systematic sequential order of KCs across the cortex. This was achieved by repeating the analysis described above after the addition of the template KCs, where a multinomial test was used to identify channel pairs with significant sequential ordering. Due to the addition of so many more KCs, a total of 412 pairs of channels (compared with 341 pairs in the manual-only KCs) were tested over a representative night for all nine patients. Of these pairs, 33 pairs over four subjects were significant in addition to the pairs found using only manual KCs, for a total of 53 significant pairs (13%). Subjects 1, 4, 8, and 9 did not show any significant pairs before or after the addition of KC-like activity. A single significant pair, from ventral to dorsal, was found in subject 5 after the addition of KC-like activity. Subject 6 did not add any additional pairs.

Subject 3 was one of the subjects for whom anterior-to-posterior propagation could be most clearly tested due to electrode placement in prefrontal and parietal cortices (Fig. 7*A*). Of six prefrontal–parietal pairs examined, there were no significant pairs using only manual KCs, and, even after the addition of KC-like activity, there was no evidence of significant frontal-to-parietal propagation; rather, there were two significant pairs found within frontal cortex. Subject 7 did not have any significant manual pairs; however, one pair along the same electrode became significant after the addition of KC-like activity. Subject 2 already showed a large number of significant pairs (16) in testing manual KCs; the addition of KC-like activity added 29 significant pairs (Fig. 7*B*). This subject shows a strong tendency for significant pairs, in fact 64% of such pairs, to lead to temporal cortex. Apart from this pattern, the other pairs show sequences in many directions within and between hemispheres. In addition to the 47 significant pairs plotted in Figure 7, there are only 6 other significant pairs over three subjects, including both manual and KC-like activity, that were significant and have been described above but are not plotted. While the addition of KC-like activity makes it more possible to see evidence of anterior-to-posterior propagation, frontal-to-parietal propagation still is not seen in the eight contact pairs in three subjects where it could be tested. Although propagation within the frontal lobe in various directions is seen, the only consistent propagation between lobes is from frontal to temporal.

### Insular origin of KCs is not supported by intracranial recordings

In addition to the channel pairs that had a significant order of KC occurrence, we examined a specific prediction made in the literature regarding the typical origin of KCs. Murphy et al. (2009) interpreted scalp EEG as demonstrating that 46% of isolated SOs (i.e. KCs) originate in the insula. Using bipolar SEEG, we demonstrated insular generation of KCs in two contacts in two patients. Of 1080 KC events in these subjects, only 177 (16%) involved the insula. Thus, at least 84% of KC events originated outside the insula. Of the KC events that did include the insula, not all began in the insula. Specifically, of the 61 KC events involving the insula and at least one other structure, the insula led in 38 KCs (62%). If we extrapolate this proportion to all insula KCs, then the proportion of all KCs led by the insula would be ∼10% (62% of 16%). While our sampling of two insular contacts from two patients is small, the inference of Murphy et al. (2009) does not appear to be supported by direct intracranial recordings.

### KCs may show a slight anterior-to-posterior propagation when all individual trials are tested

**Figure 11.**
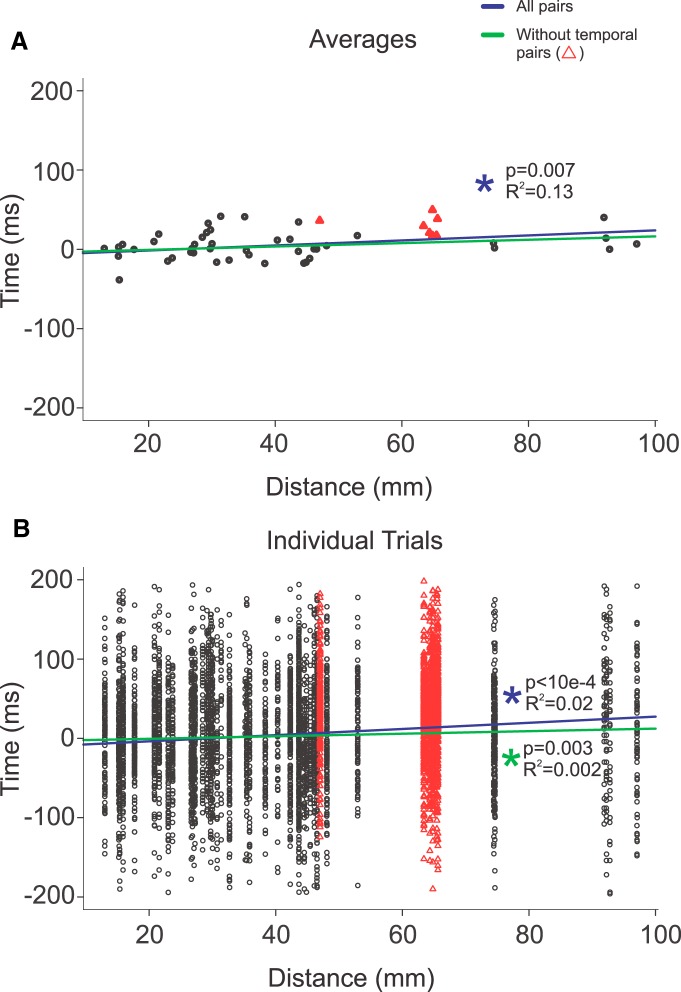
Anterior-to-posterior contact pair distance versus KC latency delay. ***A***, The distance between anterior-to-posterior channel pairs was plotted as a function of the average KC latency difference between the two channels, for all contact pairs (black circles and red triangles). The linear mixed-model regression between anterior-to-posterior distance and delay for all pairs is plotted as a purple line (*p* = 0.007), and, after excluding the temporal pairs (plotted with red triangles), is plotted as a green line (*p* = 0.08). ***B***, The distance between the same anterior-to-posterior channel pairs as in ***A*** was plotted as a function of each individual KC latency difference (black circles and red triangles). A linear mixed-model regression calculated to determine whether a significant relationship existed for individual KCs on these pairs was significant at *p* < 10^−4^ (purple line). Excluding the temporal pairs plotted with red triangles, the linear mixed-model regression was still significant at *p* = 0.003 (green line).

In order to directly test the hypothesis that there is a significant distance versus delay relationship between anterior and posterior cortex during the KC, we examined 6224 manual plus template-detected KCs that involved 51 pairs located along the anterior-to-posterior axis. To isolate anterior-to-posterior propagation from lateral-to-medial propagation, selected pairs could only have one contact separation in the medial-to-lateral direction. We found 3702 KC pairs where the peak latency occurred first in the anterior channel followed by the posterior channel; in contrast, we found only 2518 KC pairs that showed the opposite progression from the posterior to the anterior channel. This represents an ∼47% increase of KCs in the anterior-to-posterior direction compared with the posterior-to-anterior direction. The remaining four pairs exhibited a 0 ms delay. The distance between these channel pairs was plotted as a function of the average KC latency difference between them (Fig. 11*A*, black circles and red triangles). Distance between channel pairs was also plotted against the individual KC latency differences between them (Fig. 11*B*, black circles and red triangles). A linear mixed-model regression found that a significant relationship between anterior-to-posterior distance and delay existed across these pairs, considering either the average KC latency difference (Fig. 11*A*, purple line; *p* = 0.007) or the individual KC latency difference (Fig. 11*B*, purple line; *p* < 10^−4^).

As we found that a large number of pairs in one subject exhibited a significant propagation from prefrontal areas to temporal cortex, we examined the effect of eliminating these pairs, which are plotted as red triangles in Figure 11. When these pairs are excluded, there are 2394 pairs that progress from anterior to posterior and 2061 pairs that progress from posterior to anterior, which only represents a 16% increase in anterior-to-posterior pairs. The black circles in Figure 11 represent the nontemporal pairs that were included in a subsequent linear mixed-model regression, which did not find a significant relationship between anterior-to-posterior distance and average KC latency differences (Fig. 11*A*, green line; *p* = 0.08); however, when KC latency differences were considered individually, a significant relationship again occurred (Fig. 11*B*, green line; *p* = 0.003).

In all cases, significant distance versus delay relationships explained only a small percentage of the variance: 2% when all pairs are considered individually, 13% when the average KC latency differences for all pairs are used, or 0.2% when individual pairs, excluding temporal pairs, are considered.

The above analyses considered the relative anterior–posterior location of the two contact pairs, in order to test the hypothesis that there is a global anterior-to-posterior propagation of KCs. This hypothesis predicts that anterior sites would precede posterior sites, and that the time delay would increase with increasing distance. We also tested the hypothesis that, regardless of direction, there is an overall relationship of distance versus delay, by examining the absolute distances and absolute delays of all pairs. As the distribution is no longer normal, a regression could not be calculated. Instead, the correlation calculated between the absolute distance and absolute delay of these KC pairs was very small (*r* = 0.071), although it was significant (*p* = 2.1e-08). As a direct comparison, the correlation was also calculated between the signed distance and absolute delay, as modeled above. This correlation was again significant, and greater than that for absolute distance and absolute delay, but it was still small (*r* = 0.12, *p* < 2.2e-16).

In summary, there is a highly significant excess of KCs that propagate in the anterior-to-posterior direction compared with the posterior-to-anterior direction, and the average delays between locations are related to the distance between them, provided that the frontal-to-temporal pairs are included. However, although this is true on a statistical basis, the variation between sites and individual KCs is very large, so this effect only explains a very small part of the observed pattern.

### Stage 2 and stage 3 KCs have similar neurophysiological characteristics

In the above analyses, KCs were defined by their morphology and accompanying decrease in high gamma power, and thus were not confined to stage 2 sleep. In order to compare NREM stage 2 sleep (N2) and N3 KCs, sleep staging was performed on scalp electrodes for the patients from Massachusetts General Hospital and on SEEG for the patients from La Timone Hospital, where scalp EEG was unavailable. Unless otherwise noted, all analyses presented below were performed on the combined manually detected and template-detected KCs. There were 2.8% fewer KCs added using template detection during N2 than N3, which was not significantly different when tested over all subjects using a paired *t* test (*p* = 0.64).

**Figure 12.**
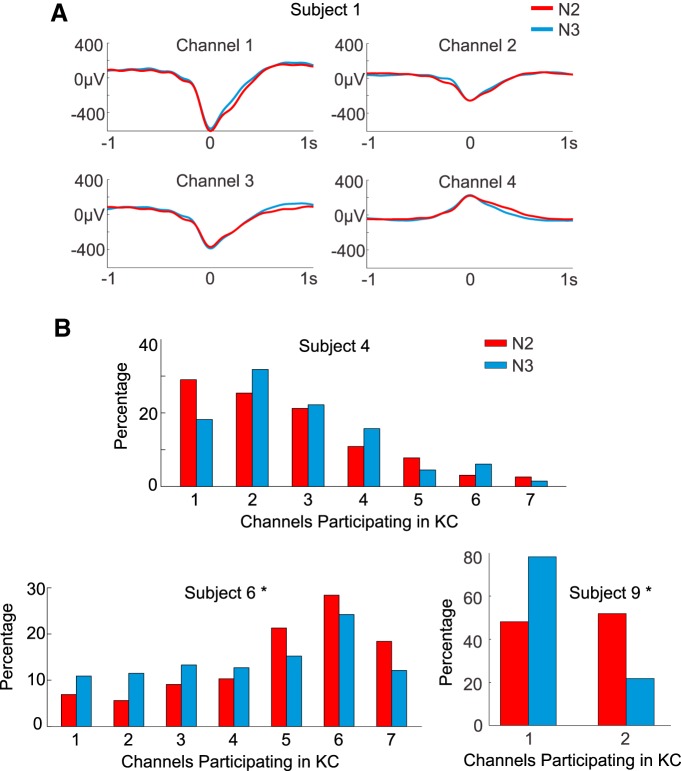
Comparison of N2 and N3 KCs. ***A***, Waveforms of averaged N2 and N3 KCs overlaid for subject 1’s four channels exhibit highly similar KC morphology. ***B***, Multichannel KC participation is not consistently greater in N2 or N3. The percentage of KCs occurring in each number of channels for N2 (red) and N3 (blue) are plotted for subjects 4, 6, and 9. Subjects 6 and 9 show N2 KCs involving more channels, but this is not the pattern seen in all subjects, as shown with subject 4. When a χ^2^ test was performed for each subject on these distributions, only subjects 6 (*p* = 0.011) and 9 (*p* = 0.000056) exhibited significant differences.

Averaged KC waveforms overlaid for N2 and N3 for the four channels of subject 1 have indistinguishable morphologies (Fig. 12*A*). To test whether there is a statistically significant difference between N2 and N3 peak amplitudes, *z*-scores for N2 and N3 were calculated for the KC peak amplitude for each channel with respect to the null values. When tested, *z*-scored amplitudes for N2 and N3 over all channels show no significant difference (paired *t* test, *p* = 0.9).

To evaluate whether a difference in the high gamma drop that characterizes the down state differs between N2 and N3, the Hilbert analytic amplitude for high gamma power was calculated from 25 ms before to 25 ms after the KC peak at 0 ms for all KCs. These values were averaged over all KCs in a channel, separately for N2 and N3. A paired *t* test between the averaged N2 and N3 high gamma values over all channels found no significant difference (*p* = 0.34).

In order to determine whether manually marked N3 KCs more closely resemble a slow oscillation than N2 KCs, the pre-KC peak amplitude was detected within a −850 to −350 ms window prior to the KC peak at 0 ms and compared with the amplitude of this KC peak. Assuming that the up and down states of a slow oscillation have the same amplitude but opposite polarities, a slow oscillation would be implied if the pre-KC peak were equal to the KC peak. This was a more direct measure of whether the KC was preceded by a slow oscillation up state than the previously used pre-KC variance measure, which tested overall variability and was not scaled to the expected value of an SO. When the means over all KCs over all channels are calculated, the pre-KC peak of both manual N2 and N3 KCs is equal to 26% of the KC peak; in comparison, template-detected N2 KC means are 67%, while template-detected N3 KC means are 83%. If the mode is calculated instead, pre-KC amplitude of manually detected N2 KCs are 19% of the KC peak, compared with 22% for manually detected N3 KCs; again, this is much smaller than for the template-detected KCs, which are 43% for N2 and 44% for N3. Therefore, the manually detected KCs showed similar pre-KC amplitudes regardless of sleep stage, which were much smaller than would be expected for a slow oscillation and smaller than template-detected KCs.

To test for a difference in the occurrence rate between N2 and N3, the average interstimulus (i.e. KC) interval for each stage for each channel was calculated after applying a Box-Cox transform to normalize the data. Of all 55 channels, 32 channels (58%) occurred less frequently for N2 than for N3. A one-tailed paired *t* test found that N2 KCs occur less frequently than N3 KCs (*p* = 0.003). In summary, KCs in stage 2 and 3 NREM sleep have very similar morphologies, pre-KC amplitudes, KC peak amplitudes, and accompanying decreases in high gamma power. N3 KCs occurred more frequently than N2 KCs.

### KCs in N2 and N3 do not differ in the number of participating channels

A separate χ^2^ test was performed for each subject, comparing the number of KCs occurring in N2 and N3 over each number of channels participating in a KC (i.e., one channel up to the maximum number of channels recorded for that subject). For the majority of subjects, there was no significant difference between N2 and N3 KCs in terms of multichannel participation, as follows: subject 1 (*p* = 0.055); subject 2 (*p* = 0.077); subject 3 (*p* = 0.068); subject 4 (*p* = 0.061); subject 5 (*p* = 0.78); subject 7 (*p* = 0.97); and subject 8 (*p* = 0.38). While some of these values are close to reaching significance, there is no clear pattern of one stage involving more or fewer channels than the other stage. As an example of this, subject 4 is presented in Figure 12*B* with the percentage of KCs occurring in each number of channels by stage, with N2 plotted in red and N3 in blue. For two subjects, there was a significant difference between the distribution of channels participating in N2 and N3 KCs [subject 6 (*p* = 0.011), subject 9 (*p* = 0.000056)]. In both cases, the distribution of N2 KCs is skewed to the right, indicating that N2 KCs involve a greater number of channels than N3 KCs for that subject. The percentage of KCs occurring in each number of channels by stage for these two significant subjects is plotted in Figure 12*B*.

These results could indicate that there is a greater co-occurrence of a KC across locations during N2 compared with N3. Subject 9, however, only involved two channels, and therefore, it is difficult to predict whether this relationship would remain with the addition of more channels. The results for the other subjects that did not reach significance also exhibited variable patterns, some with slightly more multichannel KCs in N2, and others in N3, or neither. Thus, if there is a difference between stages in multichannel participation, it is weak.

### KCs in N2 and N3 do not differ significantly in their propagation sequences

As described above, a significantly consistent direction of propagation between each pair of bipolar recordings was detected using binomial tests. Since the power of the binomial test is critically dependent on the number of observations, we subsampled the N2 or N3 data in any given patient so that the number of KC events was equal for the two sleep stages. With the much-reduced power, the number of significant pairs was only 39 for N2, and 27 for N3, without Bonferroni correction, of 180 bipolar pairs (χ^2^ = 2.67, df = 1, *p* > 0.10). Due to the small number of remaining events after balancing N2 and N3 in each subject, we are unable to draw any strong conclusions regarding whether the patterns of sequences are significantly different in N2 or N3.

In summary, the similarities and differences between N2 and N3 KCs support the hypothesis that physiological and morphological properties define the KC, rather than their occurrence during a particular stage. For the majority of subjects, there was no difference in multichannel participation between stages, while findings regarding propagation patterns were inconclusive. We conclude that the defining physiological and morphological characteristics of a KC do not differ between N2 and N3, and therefore, that global KC properties may be studied collapsed between stages.

### Summary of current results

**Figure 13.**
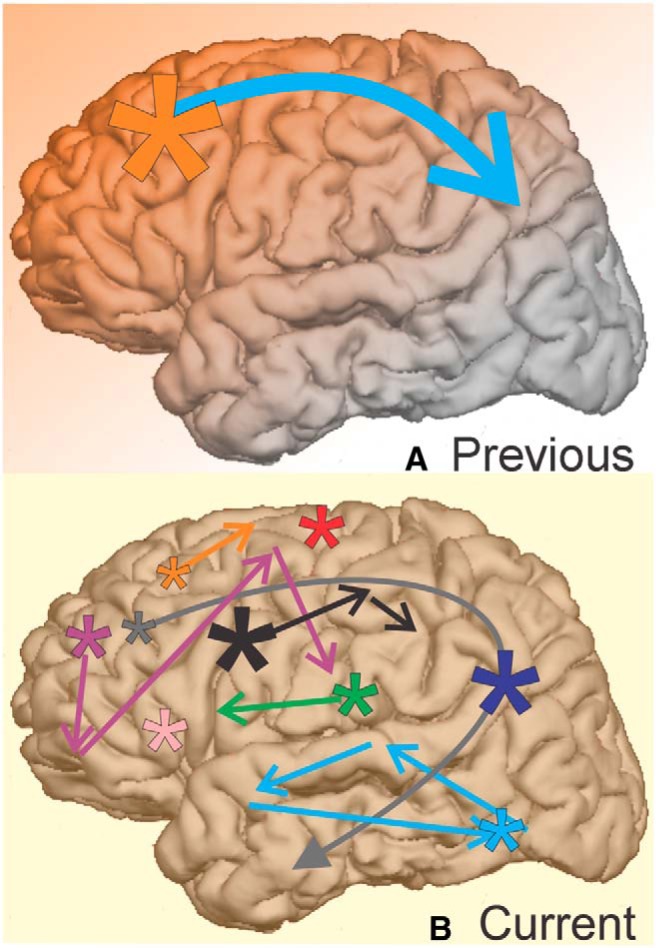
Summary of current KC findings compared with previous views. ***A***, Previous scalp EEG and intracranial studies of the KC and isolated SOs (i.e., KCs) find the KCs with the largest amplitudes in midline prefrontal regions (orange star), as well as strong (blue arrow), one-directional (faded orange background) propagation from anterior to posterior cortical regions. ***B***, Our current findings indicate that when measured locally, KCs are actually smallest in amplitude in anterior prefrontal regions (orange star). KCs may co-occur (uniform yellow background) across variably small or large areas of cortex (stars and arrows). Propagation may occur from anterior to temporal regions (gray arrow), but also occurs in a variety of patterns across the cortex (groups of colored arrows). This variability in KC onset, extent, and spread could be used by the cortex during replay to consolidate memories encoded with correspondingly variable spatiotemporal encoding patterns.

Figure 13 depicts a summary of our findings in contrast to current views. Based on scalp EEG and MEG, as well as referential intracranial recordings, a monolithic interpretation of KCs (or their equivalent isolated SOs) has been put forth wherein KCs are generated initially and most strongly in prefrontal cortex (Fig. 13*A*, orange star), and then propagate consistently to parietal cortex (Fig. 13*A*, blue arrow and fading orange background). In contrast, our results demonstrate that KCs are smallest in anterior prefrontal areas (Fig. 13*B*, orange star), that they arise all over the cortex (additional stars), and that they may co-occur across many areas, as represented by the uniform yellow background. While there is a weak overall tendency for KCs to propagate posteriorly, especially from frontal to temporal sites (gray arrow), KCs seldom exhibit a consistent pattern of propagation between any two cortical locations (groups of colored arrows). Furthermore, the current results show that while some KCs involve widespread cortical locations, others can be very focal. In summary, our results suggest that each KC may be unique in its origin, extent, and spread.

## Discussion

Although KCs have been studied for over 80 years, their origin and spread remain controversial. Do they occur quasi-synchronously in widespread cortical areas (Mak-McCully et al., 2014), or arise in midline frontal areas and spread parietally (Massimini et al., 2004)? Are KCs a fundamental cortical process, or are some cortical areas unable to generate KCs (Wennberg, 2010)? In locations generating KCs, are their amplitudes and occurrence rates uniform? These issues remain controversial because previous studies used methods that provide ambiguous localization. We examined the spatial and temporal relationships of individual KCs recorded simultaneously in multiple cortical locations using bipolar SEEG recordings, validated with HGP. Using this unambiguous method, we found that KCs can be generated throughout the cortex, including areas previously asserted not to generate KCs: the cingulate, ventral temporal, and occipital cortices. Contrary to previous accounts, KCs were smallest in anterior prefrontal areas. A given KC may occur in only one sampled location, or may co-occur in widespread cortical sites. When KCs were absent in other channels, KC-like activity was often found. Regardless of whether this KC-like activity was included, most channel pairs did not show a regular propagation direction. However, there was a weak statistical tendency favoring anterior-to-posterior propagation. We conclude that KCs have multiple degrees of freedom that may support the spatiotemporal patterns underlying memory consolidation and cortical restoration.

### Methodology for obtaining unambiguous generator localization

Previous examinations of KCs in humans used EEG, MEG, EEG with fMRI, ECOG, or referential SEEG. In contrast, our study used bipolar SEEG derivations, where one contact was immediately above the local cortical surface, and one was in the immediately subjacent white matter. We validated KCs by demonstrating an accompanying statistically significant drop in HGP. Thus, unlike previous studies, we could be certain of local generation at the electrode site.

Dense microelectrode array recordings demonstrate that the KC is a cortical down state, with a cessation of neuronal firing and a broadband decrease in spectral power of the local field potential (LFP), due to inward apical dendritic currents in layers 2/3 (Cash et al., 2009). The current return is in layer 1, resulting in a surface negativity in ECOG or SEEG. Ambiguities arise because each sensor records the summation and cancelation of all cortical potentials. Although distant areas contribute to the LFP inversely according to the square of their distance (Lindén et al., 2011), their total contribution may exceed that of local sources when much of the cortex is active (Klee and Rall, 1977).

All potential measurements are relative to a reference electrode, and the above comments assume an inactive reference. However, when generators are distributed, it is difficult to confirm that a reference electrode location is inactive. Extracranial references cannot be assumed to be inactive because the amplitude of distributed generators declines little from cortex to scalp (Cooper et al., 1965). Average references are not neutral unless all sides of the volume conductor are equally and densely sampled, a practical impossibility due to the neck and face, and to scalp incisions. Thus, even locally recorded potentials with ECOG and SEEG are difficult to interpret as definitively reflecting only locally generated activity.

Bipolar transcortical SEEG derivations are insensitive to distant sources because the two contacts act as references for each other. The positive and negative peaks of the locally generated KC are determined by the separation between the generating transmembrane inward and outward currents, measured to be separated by ∼1 mm (Cash et al., 2009). The two leads in the bipolar SEEG pair were separated by 2.6–5.6 mm, enabling them to capture the entire local field. In contrast, volume-conducted potentials from distant generators change little between the leads, and thus are removed by recording the difference between the leads. In addition, our KCs were accompanied by a suppression of broadband HGP, which has been observed to be highly focal (Lachaux et al., 2005), due to asynchronous generators (Lindén et al., 2011). Thus, the co-occurrence of HGP suppression provides a strong confirmation of local KC generation.

The ambiguities resulting from the superposition of volume conducted potentials from many locations is much more severe for extracranial (scalp) EEG, where all locations are distant and the intervening skull and subcranial CSF further spread the potential (Ahlfors et al., 2010; Irimia et al., 2012). Inverse solutions use the pattern of potentials across sensors to infer possible generators. However, the inverse problem is mathematically ill posed unless unproven assumptions are made. Scalp EEG also has a reference that is likely to be active. MEG does not use a reference, and is not smeared by the skull or CSF, but it also suffers from an irretrievably ambiguous inverse problem due to superposition of multiple sources at the sensors (Liu et al., 2002). While PET scanning and fMRI provide unambiguous localization, and can be triggered by KCs recorded by simultaneous EEG, they lack the temporal resolution to determine whether the activity they locate is due to the KC itself or to other time-locked activity.

Slow oscillations, and to a limited extent, KCs have also been examined in animals. SOs occur synchronously over long distances in anaesthetized (Volgushev et al., 2006) and naturally sleeping cats (Destexhe et al., 1999), as do KCs (Amzica and Steriade, 1998). SOs are also synchronous in anesthetized rats (Isomura et al., 2006). In contrast, SOs in ferret slices propagate slowly (Sanchez-Vives and McCormick, 2000). Besides the effects of anesthesia and the loss of synaptic connections in slices, the brain volume of humans is ∼750 larger than rats and ∼45 times larger than cats (Haug, 1987), making the problem of synchronization or propagation different.

In summary, the recordings reported here better characterize KC origin and spread because they were less prone to the ambiguities inherent in previous methods. However, electrodes were placed for localizing seizures in patients with long-standing epilepsy. Epilepsy can be caused by many factors, including a widespread genetic alteration in excitability that could affect the occurrence, amplitude, or spread of KCs. Conversely, the epilepsies of the patients studied here were localized and nongenetic. Rarely, KCs have been noted to be altered in partial epilepsy: KC rate increases, and elevated amplitude KC-like phenomena precede some seizures in refractory nocturnal frontal lobe epilepsy (Si et al., 2010); however, none of the patients analyzed here have this type of epilepsy. KC-like phenomena have occasionally been associated with epileptiform spikes in temporal lobe epilepsy (Geyer et al., 2006), but would have been identified and eliminated from the KCs studied here. Furthermore, we minimized the possible confounding effects of epileptiform activity by excluding all contacts in regions implicated in the subject’s epilepsy, and all epochs showing any signs of epileptic activity. Nonetheless, we cannot completely exclude the possibility that the patient’s epilepsy affected our results.

Since clinical considerations dictated electrode placement, we were also limited in the cortical areas sampled, which differed in number and location between patients. Recordings also differed in electrode contact spacing and the geometric relationship of the contact to the local anatomy. This variability was addressed with linear mixed-effects models, which assume no interaction between patient and electrode location. If a patient’s epilepsy type were to differentially influence the size of KCs in different locations, then this assumption would be violated. Overall, dense sampling in a larger number of patients would further disentangle the multiple influences on KC origin, amplitude, occurrence, and spread.

### KC generator localization

Previous studies of KC generation have yielded a variety of localizations. Inverse solutions applied to MEG or scalp EEG estimate KC generators to deep locations in the parietal lobe (Lu et al., 1992), the sylvian fissure (Iramina and Ueno, 1996), midline structures (Murphy et al., 2009; Wennberg and Cheyne, 2013), or deep in the parietal lobe or other variable deep locations (Numminen et al., 1996). These results are often inconsistent with each other and, overall, with our results. For example, consistent with our findings, Wennberg and Cheyne (2013) estimated KC generators to subcallosal or cingulate cortices. However, they are only two of many generators, EEG and MEG estimates did not agree, and other generators localized in this study were clearly incorrect, as they acknowledge. Murphy et al. (2009) estimated the scalp EEG isolated SO down state (i.e., KC) generators mainly to the anterior cingulate, with secondary inferior frontal gyrus, precuneus, and insular generators, and no significant generation in many other locations, which we demonstrate to generate KCs. The most parsimonious solution for most inverse methods when applied to widespread generators is to estimate a relatively strong but focal deep source.

Tones that evoke KCs also evoke increased BOLD in the superior temporal plane and inferior frontal gyrus; pars opercularis (Czisch et al., 2009; Dang-Vu et al., 2011). However, these increases are likely to represent BOLD activity related to the tone, rather than the KC, which is a down state. During SOs, PET scanning also shows a superior temporal increase, with a subcallosal decrease (Dang-Vu et al., 2005). While the PET scan findings are during SOs, their location corresponds with some of the areas where we demonstrated KC generation.

Wennberg (2010) investigated KC localization using average reference ECOG and SEEG values in humans, and recorded the KCs with the largest amplitude in dorsal frontal areas, which he interpreted as being the site of the largest KC generators. We demonstrate how average reference SEEG may detect KCs in locations where they are not generated. In these sites, KCs are not seen in bipolar SEEG, and they do not correspond to a local drop in HGP. Thus, the presence or absence of KCs in referential SEEG must be interpreted with caution. In contrast, we found that KCs in anterior prefrontal regions are small and infrequent, while KCs occur more frequently in posterior prefrontal cortex and posterior cingulate. We also demonstrate that KCs are generated in the cingulate, ventral temporal, and occipital cortices, where Wennberg (2010) concluded that KCs are not generated based on his failure to record them. Our data regarding ventral temporal sites are not definitive because they were located in regions implicated in epileptogenesis. The emphasis of Wennberg (2010) on dorsolateral prefrontal generators may also have partially arisen from his averaging of SEEG KCs at their frontal scalp EEG peak, while we identified KCs individually at each cortical site.

Nir et al. (2011) demonstrated that unit firing is correlated with SOs recorded in the medial superior frontal gyrus, precuneus, anterior and posterior cingulate, entorhinal cortex, and parahippocampal gyrus, thus demonstrating local generation. Although not specifically reported, their data suggest that these locations also generate KCs. Using linear microelectrode arrays, Cash et al. (2009) reported local transmembrane currents during KCs in prefrontal, lateral orbital, middle and superior temporal, and supramarginal gyri. Using the same method, Csercsa et al. (2010) demonstrated local generation of SO in the postcentral and prefrontal cortices. These studies are consistent with our finding of locally generated KCs in prefrontal, medioventral temporal, cingulate, orbital, and supramarginal cortices, and demonstrate or imply generation in the superior temporal and postcentral gyri, and the precuneus, where we failed to record. Overall, our findings, combined with those of earlier studies, demonstrate very widespread KC generation.

This conclusion is inconsistent with the studies described above, which have been interpreted as indicating that large areas of cortex do not generate KCs. Using the more definitive methods described here, every location that was adequately sampled generated KCs, implying that KCs may represent a fundamental operating mode of human cortex. This conclusion raises the question of why KCs show significantly different amplitudes in different cortical locations. A complete answer cannot be given until the precise circuit and channel mechanisms of KC generation are better understood. However, different cortical areas vary considerably in their levels of particular ligand- and voltage-gated channels, and associated proteins (Zilles et al., 2002; Hawrylycz et al.,2012), in particular, GABA_B_ and potassium channels, which may be involved in KC generation (Cunningham et al., 2006; Kohl and Paulsen, 2010).

### KC propagation

Prior to this study, there had not been a rigorous examination of how KCs may propagate across the human cortex. In an influential study, Massimini et al. (2004) proposed that scalp EEG SOs in more posterior scalp sites peaked progressively later, with a delay of ∼200 ms, or a mean propagation speed of 2.5 m/s. This view was extended by Murphy et al. (2009) in their analysis of isolated spontaneous SOs (i.e., KCs). Wennberg (2010), however, noted that KCs appeared to occur synchronously; when tested explicity (Mak-McCully et al., 2014), the observed delays in the peaks of averaged KCs between anterior and posterior sites in EEG, ECOG, or SEEG were ∼10 times faster than had been previously predicted for isolated SOs (Massimini et al., 2004; Murphy et al., 2009). Since averaging could obscure propagation patterns, we systematically tested here for such patterns in single KCs. Again, we found scant evidence for regular propagation patterns. When KCs occurred on different bipolar contacts within 200 ms of each other, they were tested to determine whether their order of occurrence was significantly different from chance. This occurred in only 6% of manually detected KCs, or 13% when template-detected KCs are also included. Several cases of significant propagation were observed within the frontal lobe, including along the cingulate, where propagation could be in any direction. Such complex patterns of propagation across the cortex have previously been noted by Hangya et al. (2011). Frontal-to-parietal propagation, however, occurred in none of the eight pairs of bipolar contacts where it could be tested. A preponderance of insular origins of isolated SOs (i.e., KCs; Murphy et al., 2009) was also not supported by our data in the two patients in whom it could be tested. The most common pattern of propagation was from lateral prefrontal to lateral temporal, which is reminiscent of the regular SO propagation from medial frontoparietal to medial temporal lobe locations found by Nir et al. (2011), who also failed to find regular propagation within the medial frontoparietal sites. SO propagation from anterior to temporal regions for both medial and lateral cortex was also found by Botella-Soler et al. (2012).

Notwithstanding these negative results, we found a highly significant excess of KCs that propagate in the anterior-to-posterior direction compared with the reverse, and that average delays between locations are related to their spatial separation. Although this is true on a statistical basis, the variation between sites and individual KCs is large, so this effect explains only 2% of the observed pattern.

### KCs are not confined to stage 2

Sleep stages defined using scalp EEG do not necessarily indicate a uniform and synchronous participation by different brain areas. For example, sleep onset measured locally in the thalamus has been noted to occur before sleep onset is detected in scalp EEG (Caderas et al., 1982; Gücer et al., 1978) or locally in the cortex (Magnin et al., 2010). We have also observed that isolated KCs may occur in parietal areas, while rhythmic slow oscillations occur in frontal areas. Thus, we rigorously detected locally generated KCs using LFPs and HGP, and then characterized them regardless of sleep stage. *Post hoc* analyses showed that pre-KC amplitude, KC peak amplitude, and high gamma characteristics did not differ between stages 2 and 3. However, KCs did occur more frequently in N3 than N2. Critically, the extent of KC cortical involvement does not appear to change between stages.

### Functional implications of KC heterogeneity

In summary, this is the first study to not only characterize the local generation of KCs in human cortex, but to examine their variable amplitude and occurrence rate, co-occurrence across the cortex, and propagation (Fig. 13). Although systematic propagation of KCs between cortical sites can be observed, these are rare, and, in most cases, the patterns of propagation vary randomly from KC to KC. Similarly, although there is a slight overall tendency for more anterior sites to lead more posterior ones, this tendency only explains a minute proportion of the variance, again leading to the conclusion that successive KCs are highly heterogeneous. We show in this article that KCs differ greatly not only in their propagation patterns, but also in which areas are engaged, even though all areas seem to generate some KC activity. It has previously been shown that KCs can be universal and quasi-synchronous across widespread cortical sites (Mak-McCully et al., 2014). Here, we confirm this finding and show that KCs can also be highly focal or intermediate. This conclusion was robust to different methods of clustering KCs across channels, and was strengthened when using template-selected as well as manually selected KCs.

KC heterogeneity may arise from different cortico-cortical or cortico-thalamic mechanisms underlying the KC. Recent work has re-examined the role of the thalamus in the production of SOs, which have long been thought to be an exclusively cortico-cortico mechanism, and found the thalamus to be important for SO frequency (David et al., 2013) and synchrony (Lemieux et al., 2014). Additionally, disruption of spindling in the thalamus has been posited as a mechanism of quasi-synchronous KC generation (Mak-McCully et al., 2014). Variable involvement of thalamic versus cortical mechanisms may help to account for variability in the location and spread of KCs.

KC heterogeneity may also reflect differential engagement of different cortical areas during the preceding waking period (Huber et al., 2004). This modulation of KC distribution may reflect restorative or consolidation processes during sleep. The later function is supported by the correlation of increasing SOs with behavioral consolidation (Marshall et al., 2006). In rodents, KCs are associated with the replay of firing patterns in the hippocampus and neocortex (Ji and Wilson, 2007). The previous conception of KCs as monolithic in origin, extent, and spread would offer little scope for KCs to support the consolidation of distinct memories. In contrast, our demonstration that individual KCs are highly variable in location and propagation could provide a substrate for memory consolidation processes to engage different cortical networks, or the same networks in different sequences.

## Synthesis

The decision was a result of the Reviewing Editor Liset Menendez de la Prida and the peer reviewers coming together and discussing their recommendations until a consensus was reached. A fact-based synthesis statement explaining their decision and outlining what is needed to prepare a revision is listed below.

Both reviewers agree your work advances the field and found the ms interesting. An overall strength of the ms is the exhaustive analysis of K-complexes recorded intracranially in humans to an extent that has not been performed in a comparable manner before.

However we all found there are some critical issues that may affect some of your conclusions and that need to be carefully addressed. R1 states that data heterogeneity reflecting a variety of recording setups with different electrode types, sampling rates, electrode localization, etc... may affect quality. For R2 the focus on isolated K-complexes (KCs) may explain differences with prior work and recommends including rhythmic KCs (i.e. the slow oscillation). In addition, we feel there are some potential confounding factors (sleep phases, sampling issues and the epileptic condition) that you need to further clarify.

R2 recommended rejection. This reviewer considers that the workload would eventually require more than 2 months. However, for R1 and me, we feel you should take the chance and therefore we all have agreed to grant you with a decision to revise and re-review.

Critical major issues for us are the following:

- Issues regarding sleep phase dependence (R1 and R2' major concerns), the sampling problem (R2's general comment and my point 2) and epileptic condition (my point 1). All this should be thoroughly addressed.

- Signal definition and bipolar configuration: This is touched by R2 (major point in Lines 277-278) and by myself (my point 3 below)

I copy below the report of your reviewers and my own notes on an independent reading of your ms. If you chose to resubmit, all of our concerns should be addressed in your rebuttal letter.

A note regarding my suggestion for reorganization of figures 7 and 8 (my point 11): we wonder whether a figure for amplitude distribution is really informative given that amplitude is arbitrarily defined in your bipolar configuration (see R2's comments on this regards).

-----

R1's comments:

In this manuscript, electroencephalographic signals from epileptic patients with implanted depth electrodes were used to analyze properties of K-complexes - a neocortical hallmark of mammalian light sleep.

The procedures described in the manuscript are complex, thought through and clearly reflect substantial effort. The data analyzed for the study are highly heterogeneous. A variety of recording setups with different electrode types, data sampling rates, electrode localization techniques, and number of channels were used to gather data in different countries from patients of highly varying age. All this has the potential to affect data quality, however, reasonable steps seem to have been taken to appropriately incorporate the recordings into one consistent analysis.

Main issues:

A serious methodological concern is the apparent lack of polysomnographic measures. I could not find any mentioning of sleep stages or even indicators that the patients were asleep during the analyzed time windows. One would expect an analysis of K-complexes to be confined to S2 sleep. Occurrences during wakefulness could indicate a flaw in the detection method while occurrences during slow wave sleep would rather suggest slow oscillations instead of K-complexes. This holds especially when drawing conclusions about the function of the detected phenomena.

Related to this: The relationship between K-complexes and slow oscillations (SOs) remains elusive in the manuscript. Why were K-complexes chosen as the target of investigation? This decision seems to be based on methodological grounds, single down-states are easier to tell apart and their propagation through the cortex is less ambiguous. If this is the case, it should be stated explicitly. In any case, as long as KCs and SOs are not deemed identical in spatial extent, physiological mechanism, and function, publications that focus predominantly (e.g. Massimini et al 2004) or entirely (e.g. Murphy et al. 2009) on slow oscillations should not be cited as investigating K-complexes.

Further Issues:

The mean age (+- sem) of the patients should be stated in the text (line 89), as well as the number of analyzed channels.

No clear amplitude threshold was used for automatic KC like activity detection. It should be explained why! Mean amplitudes per subject should be reported.

The paragraph describing the validation of the KC detection based on HGP decline (line 132ff) is hard to follow and should be refined.

It should be explicitly stated that KCs were not validated individually. Only the first night of each subject was used and Hilbert analytical amplitudes were averaged for each channel before evaluation.

In line 319, does "in this latter case" refer only to the "up-to-four-or-more" test or also the "three-or-more" test?

Results of the test for sequential order are reported Bonferroni-corrected. Since in the case of 341 tests this is quite a conservative measure I would prefer to additionally see the number of uncorrected significant tests (line 329).

In the same paragraph, please clearly separate results of the tests for sequential order and latency consistency.

The paragraph describing the analyses of amplitude and variance in the pre-KC period (line 376ff, Figure 5C) is hard to follow and I have no idea what the authors want to show here.

Colors in Figure 1, 7, 8 would profit from more contrast. Especially green and black are hard to tell apart.

Figure 8: Shouldn't the KC occurrence rates in B always be larger than the rates in A?

In several places in the manuscript a reference of "(Anonymous, 20XX)" is cited. What does this mean?!

----------------

R2's comments:

This is a very interesting study of (isolated) K-complex (KC) generation and propagation using implanted (and presumably localized) recordings in human epileptic patients. There are important data here that should be disseminated but some of the novel interpretations may go beyond the data studied and their generalizability may be suspect.

General Comments:

The overall conclusion made (lines 17-19 - abstract) is that while KCs can occur at multiple sites in cortex (i.e., that they are universal) "each KC may variably involve small or large cortical regions, and spread in variable directions". There is a question of originality and generalizability here. Local KCs (and slow oscillations: SO) have been previously shown (Huber et al, 2004; Nature 430:78), and may even arise during wakefulness (Vyazovskiy et al ‎2011; Nature 472: 443). Indeed, the presumed novelty of this study may simply be a reflection of the proscribed limitation of studying "isolated" KCs in contrast to KCs occurring in rhythmic sequences (i.e., the SO). Thus, any differences between the present study and those prior may be entirely state dependent - that is, arising from different dynamics in network cortical states between N2 and N3 stages of sleep. In this respect, it would be of substantially more interest within the design of the present study to show if this is the case by studying the similarities and differences of cortical "downstates" that are associated with isolated KCs versus those that form part of the ongoing SO. Without this, unfortunately, no broad conclusions can be drawn.

Another concern relates to recording coverage. While I appreciate that electrodes are implanted based on clinical criteria, I am concerned by the variability in number of sites across patients. Can broad conclusions be made using the inclusion of patients with only two sites that might be either adjacent or distant from one another? As well, the contribution of inter-site distance to temporal measures should be better elucidated.

Specific Comments:

(minor) Line 40: an explanation for the abbreviation SEEG is needed.

(minor) Line 75: "denied" as a word usage seems awkward and inappropriate for the desired meaning. Perhaps "disputed" or rephrase to suggest "not found"?

(major) lines 113-114: "This bipolar method is free of volume conduction and reference lead issues, obtaining unambiguously focal cortical recordings." Reference and citation needed for this important point that I agree with but needs empirical (not simply theoretical) evidence to back it up.

(minor - but see further below) Line 269: Figure 7 (and later 8) is referenced out of order. Consider reorganizing figures or results to make a better flow.

(major) Lines 277-278: "Generally, the cortical areas that generated large KCs were also the locations where KCs occurred most frequently (Figure 8A)." This is a problem that impacts on the conclusions of locality. The amplitude (and polarity as shown in Figure 4) of signals likely relates to the relative positioning of each of the individual poles of the bipolar pair of contacts. Indeed, with respect to this point, the utility of Figure 7 as an important result by itself is highly limited. However, it will impact on the ability to detect events per se. Can the signals be normalized by the RMS (or total power) of the spontaneous (state-constant) signal? Since gamma loss is used to confirm detections, can power in this bandwidth be used? Can gamma loss by itself be used as a criteria of detection? That numbers of detections can influence findings is directly supported by the data when comparing manual to template detection techniques.

(minor) Line 391: Delete "there" after "therefore"

(minor) Figure 1: Consider better labels for subject number. For example, writing out "Subject number/#" and simply enumerating colours would be more comprehensible.

(major) Figure 5A+B: is variance lower for manual detections due to the lower n?

(minor) Figure 5A: add units to pre-KC variance label.

(minor) Figure 5C: consider an expansion of data at low z-score values in order to be able to resolve.

(major) Figure 7: omit and consider the implications of amplitude on detections and the general conclusions.

(major) Figure 8: how well correlated is occurrence to average amplitude? See comment just prior

(major) Figure 10: this figure is confusing in terms of both positive and negative lags. I understand that the alignment is in anterior-posterior, but shouldn't distance and lag be related for the sequence in which they are recorded? That is, the absolute value of the time difference should be correlated with distance for assessing (presumed) propagation. It is not clear what conclusions might be drawn from the arrangement of data as shown.

----

Editor's notes

1.The epileptic condition could affect the expression of K complexes (Steriade TINS 2005; Bragin et al. Neuroscience 2012). Authors need to discuss whether obtaining their data from epileptic patients could affect their conclusions.

2.Uneven sampling of disparate regional territories could be another strong axis of variability that should be discussed

3.Authors base most of their methodological analysis in using bipolar transcortical sEEG. However the typical shape of K complexes can be distorted with such a bipolar arrangement. Since most neurophysiologists based their identification criteria in waveform recognition of common-referenced recordings, it would be useful to insert a figure showing such a transformation. Is raw data shown in Fig.2 bipolar or common referenced? Consider inserting an explanatory panel in this figure regarding transcortical bipolar transformation of common -referenced signals.

4.Consider adding anatomical information to Fig.1 to facilitate identification of the different anatomical regions mentioned in the ms (lines 254-263)

5.Line 49: the effect of common reference can be also mentioned here

6.Line 58: Consider the following not cited workfor the issue of the effect of common reference: Fein et al. 1988 Electroencephalogr. Clin. Neurophysiol. 69; Nunezx et al. 1997 Electroencephalogr. Clin. Neurophysiol

7.Line 69: Is there any suitable reference on the role of this effect?

8.Line 99-102: data was acquired with different macroelectrode design (spacing 5mm, 8 mm or 3.5 mm) resulting in different spatial sampling. Is there any effect of these parameters in event waveform after bipolar subtraction?

9.Line 105: data was acquired with different sampling frequencies. Is the analysis standardized by down sampling higher rates? Please, clarify.

10.Lines 106-114 and lines 123-131 should be probably close together. Here consider my previous comment 2

11.Lines 269 and 278: Consider moving Figures 7 and 8 early in the ms

12.Line 342: consider moving Fig.9 early in the ms

13.Lines 365-367: please, provide statistical confirmation of these differences

14.Line 391: '...therefore, there we...'

15.Lines 447-463: statistical validation should be included (for data in current Fig.7,8)

16.Lines 499: Is the insular cortex sufficiently sampled in the data base? It may be useful to provide with a table that identify the number of channels/patients for each relevant anatomical structure

17.Line 656; 'time-locked activity'? Do authors mean event-relate potentials?

18.Line 660: may be useful to make a cartoon summarizing current data and proposal including this one. Also useful to briefly discuss animals' literature which should probably consider the allometric effect of the brain size. Connect with point 1.

19.Table 2: what's the meaning of an empty cell?

20.Fig.4: why is the first line left most individual KC of a different shape as the template?

21.Fig.7 and 8, add scale circles
